# Supplementary material for: Photodynamic versus white light-guided treatment of non-muscle invasive bladder cancer: a study protocol for a randomised trial of clinical and cost-effectiveness
Source: BMJ Open. 2019 Sep 3;9(9):e022268. doi: 10.1136/bmjopen-2018-022268 (PMC6731798; doi:10.1136/bmjopen-2018-022268)
Supplement: Supplementary data [file bmjopen-2018-022268supp001.pdf]

APPENDIX-I: NICR PHOTO-T study

Sample Collection (per participant):

(a) 2 x blood samples totalling 12.5ml (10ml Streck blood collection tube for circulating DNA analysis and 2.5ml PAXgene blood collection tube for circulating RNA analysis);

(b) 2 x urine samples totalling 200ml (1 x 100ml for immediate translational processing and 1 x 100ml for biorepository storage), (c) 1 x FFPE core at baseline (plus core from recurrence, if occurs).

In total, 20 serially collected samples will be collected per PHOTO-T consented participant over the trial period (36 months), comprising 10 urine, 10 blood (5 DNA and 5 RNA) and 1 FFPE tumour tissue block. Collected at baseline (pre-treatment/TURBT) and treatment follow-up at 3, 12, 24 and 36 months or at recurrence (whichever comes first, predicted at 70% for the highest risk group over a trial period of 3 years).

Serial blood samples are requested for collection in clinic by research staff at participating investigator sites. Urine samples are provided at home by participants using specialist ‘home collection’ kits (home collection is preferable as sample quality in terms of genomic output is superior to clinically collected specimens) and FFPE blocks requested from Histopathology Departments of participating investigator sites retrospectively at the end of the trial.
