# Supplementary material for: Photodynamic versus white light-guided treatment of non-muscle invasive bladder cancer: a study protocol for a randomised trial of clinical and cost-effectiveness
Source: BMJ Open. 2019 Sep 3;9(9):e022268. doi: 10.1136/bmjopen-2018-022268 (PMC6731798; doi:10.1136/bmjopen-2018-022268)
Supplement: Author's manuscript [file bmjopen-2018-022268.draft_revisions.pdf]

BMJ Open

BMJ Open is committed to open peer review. As part of this commitment we make the peer review history of every article we publish publicly available.

When an article is published we post the peer reviewers' comments and the authors' responses online. We also post the versions of the paper that were used during peer review. These are the versions that the peer review comments apply to.

The versions of the paper that follow are the versions that were submitted during the peer review process. They are not the versions of record or the final published versions. They should not be cited or distributed as the published version of this manuscript.

BMJ Open is an open access journal and the full, final, typeset and author-corrected version of record of the manuscript is available on our site with no access controls, subscription charges or pay-per-view fees (http://bmjopen.bmj.com).

If you have any questions on BMJ Open's open peer review process please email info.bmjopen@bmj.com

BMJ Open

PHOTOdynamic versus white light-guided treatment of non-muscle invasive bladder cancer: A randomised trial of clinical and cost effectiveness

Journal:	*BMJ Open*
Manuscript ID	bmjopen-2018-022268
Article Type:	Protocol
Date Submitted by the Author:	20-Aug-2018

Complete List of Authors:	tandogdu, zafer; Northern Institute for Cancer Research<br>Lewis, Rebecca; Institute of Cancer Research<br>Duncan, Anne; University of Aberdeen Health Services Research Unit<br>McDonald, Alison; University of Aberdeen, Health Services Research Unit<br>Vale, Luke; Newcastle University, Health Economics Group, Institute of Health and Society<br>Penegar, Steven; Institute of Cancer Research<br>Shen, Jing; Institute of Health & Society<br>Kelly, John; University College London Hospitals NHS Foundation Trust, Urology<br>Pickard, Robert; Newcastle University Institute of Cellular Medicine<br>NDow, James; University of Aberdeen, Surgery<br>Ramsay, Craig; Health Service research unit, University of Aberdeen<br>Mostafid, Hugh; Hampshire Hospitals NHS Foundation Trust<br>Mariappan, Paramananthan; Western General Hospital, urology<br>Nabi, Ghulam; University of Dundee, Medicine<br>creswell, Joanne; South Tees Hospitals NHS Foundation Trust<br>Lazarowicz, Henry; Royal Liverpool and Broadgreen University Hospitals NHS Trust<br>McGrath, John; Royal Devon and Exeter NHS Foundation Trust, Urology<br>Taylor, Ernest; PHOTO Trial TMG<br>Clark, Emma; Northern Institute for Cancer Research<br>Maclennan, Graeme; University of Aberdeen, Health Services Research Unit<br>Norrie, John; Edinburgh Clinical Trials Unit, University of Edinburgh No. 9, Bioquarter<br>Hall, Emma; The Institute of Cancer Research<br>Heer, Rakesh; Northern Institute for Cancer Research

Keywords:	bladder cancer, photodynamic diagnosis, quality of life, HEALTH ECONOMICS, Cost effectiveness, Sample biorepository

SCHOLARONE™
Manuscripts

Title page

PHOTOdynamic versus white light-guided treatment of non-muscle invasive bladder cancer: A randomised trial of clinical and cost effectiveness

Authors: Zafer Tandogdu[1](*), Rebecca Lewis[2](*), Anne Duncan[3](*), Alison McDonald[3], Luke Vale[4], Steven Penegar[2], Jing Shen[4], John D. Kelly[5](₸), Robert Pickard[6], James NDow[3](₸), Craig Ramsay[3](₸), Hugh Mostafid[7](₸), Paramananthan Mariappan [8](₸), Ghulam Nabi[9](₸), Joanne Creswell[10](₸), Henry Lazarowicz[11](₸), John McGrath[12](₸), Ernest Taylor[13](₸), Emma Clark[1](₸), Graeme Maclennan[3], John Norrie[14](**), Emma Hall[2](**), Rakesh Heer[1](**)

*Authors have provided equal contribution and are joint first authors.

**Joint senior authors.

₸ PHOTO Trial Management Group – to be listed as grouped authors.

1. Northern Institute for Cancer Research, Newcastle University, Newcastle upon tyne, UK
2. The Institute of Cancer Research, London, UK
3. Health Service Research Unit, University of Aberdeen, Aberdeen, UK
4. Institute for Health and Society, Newcastle University, Newcastle upon Tyne, UK
5. University College London Hospitals NHS Foundation Trust, Urology Department, London, UK

For peer review only - http://bmjopen.bmj.com/site/about/guidelines.xhtml

6. Institute of Cellular Medicine, Newcastle University, UK
7. Basingstoke and North Hampshire NHS Foundation Trust, UK
8. Western General Hospital, Urology, Edinburgh, UK
9. University of Dundee, Medicine, Dundee, UK
10. South Tees Hospitals NHS Trust ,Middlesbrough, UK
11. Royal Liverpool and Broadgreen university Hospitals NHS Trust, UK
12. Royal Devon and Exeter Hospital NHS Trust, UK
13. PHOTO Trial patient representative, UK
14. Edinburgh Clinical Trials Unit, Edinburgh University, Edinburgh, UK

Corresponding authors:

1. Graeme Maclennan:
   - e-mail: G.maclennan@abdn.ac.uk
   - Phone:01224 438147

Key words:

- Bladder cancer
- Photodynamic diagnosis
- Quality of life
- Health economics
- Cost effectiveness
- Sample biorepository

The authors declare that they have no competing interests.

For peer review only - http://bmjopen.bmj.com/site/about/guidelines.xhtml

For peer review only

For peer review only - http://bmjopen.bmj.com/site/about/guidelines.xhtml

Abstract

Introduction: Bladder cancer is the most frequently occurring tumour of the urinary system. Ta, T1 tumours and carcinoma in situ (CIS) are grouped as non-muscle invasive bladder cancer (NMIBC), which can be effectively treated by transurethral resection of bladder tumour (TURBT). There are limitations to the visualisation of tumours with conventional TURBT using white light illumination within the bladder. Incomplete resections occur from the failure to identify satellite lesions or the full extent of the tumour leading to recurrence and potential risk of disease progression. To improve complete resection, photodynamic diagnosis (PDD) has been proposed as a method that can enhance tumour detection and guide resection. The objective of the current research is to determine whether PDD-guided TURBT is better than conventional white light surgery and whether it is cost-effective.

Methods and Analysis: PHOTO is a pragmatic multi-centre randomised controlled trial (open parallel group, non-masked, superiority trial) comparing the intervention of PDD-guided TURBT with standard white light resection in newly diagnosed intermediate and high risk NMIBC within the UK NHS setting. Clinical effectiveness is measured with time to recurrence. Cost-effectiveness is assessed within trial via the calculation of incremental cost per recurrence avoided and incremental cost per quality adjusted life per year (QALY) gained over three years, and over long term through a modelling exercise over patients' life time.

Ethics and dissemination: Formal ethics review was undertaken with a favourable opinion, in line with UK regulatory procedures (REC reference number: 14/NE/1062; Trial registration: ISRCTN84013636). If reductions in time to recurrence is associated with long term patient benefits the cost-effectiveness evaluation will provide further evidence to inform adoption of the technology. Findings will be

For peer review only - http://bmjopen.bmj.com/site/about/guidelines.xhtml

shared in lay media such as patient and charity forums and will be presented at key meetings and published in academic literature.

Article Summary:

Strengths and limitations of the PHOTO study include;

- the effectiveness of the photodynamic diagnosis (PDD) for the initial resection of intermediate and high risk non-muscle invasive bladder cancer as part of routine care will be demonstrated with a pragmatic clinical trial design,
- full-health economic evaluation will provide high quality evidence of the burden of NMIBC for the NHS
- a well-characterised trial associated biorepository of longitudinal serially collected tissue samples

For peer review only

For peer review only - http://bmjopen.bmj.com/site/about/guidelines.xhtml

1. Background

Bladder cancer is the most frequently occurring tumour of the urinary system [1]. Staging of bladder cancer is described using the Tumour, Node, Metastasis (TNM) system [2], for which an illustration is provided in Figure 1. Tumours confined to the epithelial lining (urothelium) are classified as stage Ta and those invading the lamina propria are classified as stage T1.

Ta and T1 tumours can be removed by transurethral resection (TURBT) that involves passing a cystoscope through the urethra into the bladder and resecting the tumour under direct visualisation. For therapeutic purposes Ta and T1 tumours are grouped together as non-muscle invasive bladder cancer (NMIBC). Grade (microscopic characteristics of the tumour cells) can be used to describe aggressiveness of cancers and are characterised as either low grade (relatively benign) or high grade (aggressive). NMIBC also include flat, high-grade tumours that are confined to the epithelium classified as carcinoma in situ (CIS).

Complete resection of the tumour with TURBT is essential to obtain good prognosis. It is thought that failure to identify satellite tumours or to appreciate the full extent of the tumours visualised during resection using conventional white light cystoscopy may be a factor in 20-40% of recurrent bladder tumours [3, 4]. Incomplete resection with TURBT is also associated with staging errors. In order to correct the staging errors associated with initial TURBT a second resection within 2-6 weeks is suggested for select group patients [5]. It has been postulated that development in cystoscopy imaging can improve resections and decrease the need for a second resection [6].

For peer review only - http://bmjopen.bmj.com/site/about/guidelines.xhtml

Recurrence and stage progression to muscle invasive (T2-T4) or metastatic cancer is more likely to occur in those with high-grade tumours with concomitant CIS. CIS in particular can be easily missed using conventional white light guided resection [6].

Surveillance of NMIBC is carried out with cystoscopy to detect recurrence early and allow treatment before progression. Clinical guidelines tailor follow up protocols according to the risk groups (low, intermediate and high) developed using clinical and histological parameters [7]. Advised follow-up of low risk is at three months and if negative the next cystoscopy is scheduled for nine months later and then yearly for five years. Patients with high-risk tumours have cystoscopy and urine cytology at three months. If negative, it is repeated every three months for two years, then every six months until five years, and annually thereafter [5]. The intensity of cystoscopic follow up for patients with intermediate risk is not clearly defined, for which a follow-up scheme in-between those described for low and high risk and is adapted according to personal and subjective factors [5].

1.1. Photodynamic diagnosis of NMIBC:

As an attempt to improve resection rates, photodynamic diagnosis (PDD) has been developed to enhance tumour detection and guide resection. Meta-analyses and systematic reviews of PDD guided treatment of NMIBC have shown efficacy in tumour detection and reduction in residual tumour compared with white light cystoscopy alone. These findings translate into reduced recurrence rates [6, 8]. However, these trials were efficacy studies and the systematic review called for a pragmatic study to allow better interpretation of possible benefit into daily clinical practice.

For peer review only - http://bmjopen.bmj.com/site/about/guidelines.xhtml

1.2. Health economics of NMIBC:

NMIBC is one of the most costly cancers to manage on a per patient basis because of its high prevalence, high recurrence rate, need for adjuvant treatments and the requirement for long-term cystoscopic surveillance. The total cost of treatment and 5-year follow-up of patients with NMIBC diagnosed in the United Kingdom has increased from £73 million to £213 million from 2001 to 2012 (inflation corrected) [9, 10]. From a patient perspective, there often are considerable anxieties about recurrences, transurethral resection and progression, requiring additional therapies with potential mortality and long-term morbidity (e.g. radical surgery). Transurethral resection itself is associated with reduced quality of life, including both mental and physical health domains; although these effects are usually transient [11]. Substantial effects on health related quality of life (HRQoL) are most likely to come from adjuvant intravesical treatments and radical or palliative treatments for progression [12]. The cost-effectiveness of NMIBC treatment strategies has not been widely studied.

1.3. NMIBC biomarkers and clinical impact:

To date, existing non-invasive commercial biomarkers (primarily urinary) are not embedded in routine clinical practice due to poor sensitivity, specificity and lack of evidence. Several research bodies have recognized the lack of clinically useful biomarkers for bladder cancer. “Fit for purpose” sample resources accessible to high-throughput ‘omic’ technologies will afford the greatest opportunity to generate translational hypotheses and ensure clinical validity and utility of putative candidate markers/signatures [13, 14]. Robust, ‘future-proof’, longitudinal serial sample

For peer review only - http://bmjopen.bmj.com/site/about/guidelines.xhtml

archives providing critical insights of the natural history of bladder cancer correlated with clinical detail for retrospective translational biomarker discovery, are lacking.

1.4. Current research objectives:

More efficient management strategies to reduce NMIBC recurrence and hence decrease both the burden to patients and costs are urgently needed. PDD-guided initial TURBT has been identified as a technique that can help achieve these aims. The objective of the current research (PHOTO trial) is to determine whether photodynamic surgery guided by a fluorescent tumour marker is better than conventional white light surgery in the cystoscopic treatment of people with intermediate and high risk cancers confined to the bladder lining and whether its implementation is cost-effective. The trial includes a full assessment of the costs of patient management through the care pathway. Individual patient data from this trial will be used for subsequent mathematical modelling studies to investigate safe monitoring frequency. The Photodynamic versus white light-guided treatment of non-muscle invasive bladder cancer trial has the following research objectives:

i. Primary objectives:

Clinical effectiveness: To compare time to recurrence for each of the two treatment strategies, with a principal point of interest at 3 years.

Cost-effectiveness: To evaluate cost-effectiveness as measured by the incremental cost per recurrence avoided and the cost-utility as measured by the incremental cost per quality-adjusted life year (QALY) gained at three years and over patients' life time.

ii. Secondary objectives:

a. To measure relative rates of disease progression at three years.

For peer review only - http://bmjopen.bmj.com/site/about/guidelines.xhtml

b. To measure relative harms and safety.

c. Patient lifetime HRQoL and cancer-specific survival.

iii. Additional objectives:

a. To model the safest and most cost-effective cystoscopic follow-up surveillance schedule;

b. To evaluate the learning curve for the procedure and account for its effects on outcomes of both PDD-guided and standard white light resections;

c. To establish a well-characterised cohort of serial samples from patients with intermediate and high-risk NMIBC including clinical data, urine, blood and tumour specimens for separately funded translational research.

2. Methods & Design

2.1. Study design

PHOTO is a multi-centre randomised open parallel group non-masked superiority trial comparing the intervention of PDD guided bladder tumour resection with standard white light resection in patients with newly diagnosed intermediate and high risk NMIBC. Apart from initial treatment, both groups will receive standard care, including single dose intravesical mitomycin C within 24 hours of initial resection, surveillance according to risk-adjusted schedules and adjuvant therapy as indicated by current practice guidelines. The target number of patients to be recruited is 533 with a trial specific follow-up of at least 36 months for each individual. The outline of the study protocol is shown in Figure 2.

For peer review only - http://bmjopen.bmj.com/site/about/guidelines.xhtml

2.2. Intervention

The interventions being compared within PHOTO trial are:

i. Photo dynamic diagnosis (PDD) guided trans-urethral resection (TURBT) (experimental group) vs;

ii. Standard white light TURBT (control group)

All participants, unless there are clinical contra-indications, receive intravesical mitomycin C (40 mg in 40 ml saline), ideally within 6 hours following TURBT but otherwise within the inpatient setting before discharge.

2.2.1. Technique of photodynamic diagnosis

PDD requires preliminary instillation of the photosensitiser hexaminolevulinate (85 mg in 50 ml of phosphate buffered saline) into the participant's bladder through a urethral catheter. Participants are asked not to pass urine for at least one hour after insertion. Following operating theatre preparation according to local standard procedures and under appropriate anaesthesia, participants undergo TURBT of their bladder tumour under blue light (wavelength 380-450 nm) illumination of the bladder. The equipment required includes a specialised light source, cystoscope, light cables and cameras. When using PDD, normal bladder epithelium appears blue whilst red areas should be considered suspicious and should be resected.

2.2.2. Technique of standard white light cystoscopy

The control group does not have any preliminary photosensitiser instillation and undergo standard tumour localisation and resection under white light (wavelength 400-800 nm) illumination of the bladder.

For peer review only - http://bmjopen.bmj.com/site/about/guidelines.xhtml

2.3. Participants

In the PHOTO trial adult patients with a suspected new diagnosis of intermediate or high risk NMIBC are studied. Participants are identified prior to initial resection based on results of preliminary visual assessment via cystoscopy or imaging performed as part of standard evaluation for suspected urinary tract malignancy. Patients with the following criteria are included in the PHOTO trial:

- Adult men and women aged ≥ 16 years.
- First suspected diagnosis of bladder cancer.
- Visual/ultrasound/Computerized tomography (CT) diagnosis of intermediate/high risk NMIBC.
- White light visual appearances of intermediate or high risk disease (≥3cm OR two or more tumours OR flat velvety erythematous changes alerting a clinical suspicion of CIS).

  OR

  Suspicion of papillary bladder tumour ≥3cm based on ultrasound or CT scanning (without hydronephrosis).
- Written informed consent for participation prior to any study specific procedures.
- Willing to comply with the following life style guidelines:
    - Female participants must be surgically sterile or be post-menopausal, or must agree to use effective contraception after joining the study and for 7 days after treatment. Female participants must not breast feed for 7 days after treatment.

For peer review only

For peer review only - http://bmjopen.bmj.com/site/about/guidelines.xhtml

- Male participants must be surgically sterile or must agree to use effective contraception after joining the study and for 7 days after treatment.
- Effective contraception is defined as two forms of contraception, including one barrier method.

Exclusion criteria applied in the PHOTO trial are:

- Visual evidence of low risk NMIBC (solitary tumour < 3cm).
- Visual evidence of MIBC on preliminary cystoscopy, i.e. non-papillary or sessile mass (attached directly by its base without a stalk).
- Imaging evidence of MIBC – CT/USS (this includes the presence of hydronephrosis, which may be present despite clear imaging of MIBC in the bladder).
- Upper tract (kidney or ureteric) tumours on imaging.
- Any other malignancy in the past 2 years (except: non-melanomatous skin cancer cured by excision, adequately treated carcinoma in situ of the cervix, DCIS/LCIS of the breast or prostate cancer in patients who have a life expectancy of >5 years upon trial entry).
- Evidence of metastases.
- Porphyria or known hypersensitivity to porphyrins.
- Known pregnancy (based on history and without formal testing, in keeping with day-to-day NHS practice of PDD use).
- Any other conditions that in the Principal Investigator's opinion would contraindicate protocol treatment.
- Unable to provide informed consent.

For peer review only - http://bmjopen.bmj.com/site/about/guidelines.xhtml

- Unable or unwilling to complete follow-up schedule (including HRQoL questionnaires).

2.4. Informed consent-ethics approval

Favourable ethical opinion for this research was provided by the Newcastle & North Tyneside 2 ethics committee (REC reference number: 14/NE/1062) in July 2014. The study complies with the Helsinki Declaration and the principles of Good Clinical Practice (GCP).

Potential participants are identified mainly through rapid access haematuria clinics at participating sites. An eligibility checklist is completed by the local Principal Investigator (or delegate) to assess fulfilment of the entry criteria for all patients considered for the study. Information from the diagnostic cystoscopy is used to assess eligibility.

All potentially eligible patients are provided with an information sheet to explain why they have been approached and the nature of the study. Eligible patients are asked to provide written informed consent for the study only after they have had sufficient time to consider the trial and had the opportunity to have any further questions addressed.

2.5. Recruitment and randomisation

Eligible patients are centrally randomised using either the secure web-based or the 24-hour Interactive Voice Response randomisation system at the Centre for Healthcare Randomised Trials (CHaRT) in Aberdeen, using minimisation by centre and gender, to allocate participants 1:1 to the control and experimental groups. The

For peer review only - http://bmjopen.bmj.com/site/about/guidelines.xhtml

minimisation algorithm incorporates a random element in order to prevent deterministic treatment allocation.

2.6. Outcome measures

2.6.1. Primary outcome measures

Clinical effectiveness: Time to recurrence is measured as time from randomisation to first recurrence.

Cost effectiveness: A health economic model will be developed to calculate incremental cost per recurrence avoided and incremental cost per QALY gained over three years.

2.6.2. Secondary outcome measures

Clinical effectiveness:

- Adverse events and complications up to 3 months from initial or second TURBT are captured and will be included in analysis.
- HRQOL is captured for each participant at baseline (prior to knowledge of treatment allocation), following surgery and at 3, 6, 12, 18, 24 and 36 months after randomisation.
- Disease progression is captured within the trial time. Patient life time projections will be made by using the trial data at baseline and supplementing as necessary from other published data.
- Overall survival and bladder cancer specific survival will be compared between the two treatment arms. Minimum follow-up of the last included patient will be 3 years and maximum expected follow-up is 66 months.

For peer review only

For peer review only - http://bmjopen.bmj.com/site/about/guidelines.xhtml

Cost effectiveness:

- Estimation of the incremental cost per recurrence avoided using the economic model over the patients' lifetime.
- Estimation of the incremental cost per QALY gained using the economic model over the patients' lifetime.

2.6.3. Additional outcomes measures

Schedules for follow-up: Using data from within the trial and, if appropriate, from other relevant sources, the risk of recurrence at each interval surveillance cystoscopy will be described to then model the most safe and efficient surveillance follow-up schedule.

The effect of PDD guided resection experience (learning curve) on clinical effectiveness: A subgroup analysis comparing outcomes from PDD-experienced and PDD-naïve surgeons (determined at baseline) will be conducted. Also for PDD-naïve surgeons an assessment of learning curve will be undertaken by comparing increasing experience and recurrence, in both PDD and WL resections.

2.7. Tracking and monitoring adverse events

Direct surgically related post-operative events occurring within 30 days following the first TURBT or second TURBT if required will be assessed using The Clavien Dindo classification for surgical complications. Events occurring up to 3 months after TURBT (second TURBT if required) will be assessed and recorded using the National Cancer Institute Common Terminology Criteria for Adverse Events (CTCAE) v4.0 framework (http://ctep.cancer.gov/) [15].

For peer review only - http://bmjopen.bmj.com/site/about/guidelines.xhtml

2.8. Trial assessment and measures

PHOTO trial schedule of assessment and investigations are summarised in Table 1. Routine attendances for diagnosis and staging of new bladder cancers are used to establish eligibility, which includes obtaining the medical history. Eligible patients who consent for the trial are administered HRQoL questionnaires prior to primary TURBT and prior to discharge.

Time to recurrence will be measured from the day of randomisation to the day of subsequent biopsy with pathologically proven recurrence. Data will be collected from the following routine visits; 3, 6, 9, 12, 18, 24 and 36 months post initial TURBT (or second TURBT if required). Associated costs and changes in HRQoL will be measured. These will be collected by postal questionnaires sent directly to participants at 3, 6, 12, 18, 24 & 36 months post randomisation.

Disease progression will be assessed using results of further resection or imaging during follow-up. Progression will be defined as increase of stage into MIBC or development of nodal or metastatic disease. In addition, patients showing failure to respond to intravesical treatment (e.g. BCG failure) will also be captured.

The relative changes in HRQoL resulting from the physical and psychological benefit together with any harms associated with each strategy and with subsequent necessary cancer treatment will be measured using the generic EQ-5D-3L questionnaire, cancer specific EORTC-QLQ-C30 and disease-specific EORTC QLQ-NMIBC24 questionnaire completed by the participant.

Effect of PDD guided resection experience on clinical effectiveness: All recruiting surgeons will complete a learning curve questionnaire to elicit their white light and PDD resection experience prior to any recruitment. The subsequent accruing

For peer review only - http://bmjopen.bmj.com/site/about/guidelines.xhtml

experience of each surgeon will be captured on case report forms. Early recurrence (12 weeks) will be used as a proxy of incomplete resection.

Table 1, Schedule of investigations/assessments in PHOTO trial

Visit/Assessment	Pre- randomisation screening	Pre-treatment	TURBT	Prior to discharge	Surveillance									At first disease recurrence/progression
					Second TURBT (as clinically indicated)	3 months post treatment	6 months post treatment	9 months post treatment	12 months post treatment	18 months post treatment	24 months post treatment	36 months post treatment	Annually thereafter	
Visual diagnosis of IR/HR NMIBC	X												According to EAU guidelines	Treatment according to local practice
Medical history	X													
HRQoL questionnaire[1]		X		X	X	X[2]	X[2]	X[2]	X[2]	X[2]	X[2]	X[2]		
TURBT according to treatment allocation with post treatment MMC instillation			X											
Second TURBT, if required, according to treatment allocation					X									
Assessment of adverse events (CTCAE & Clavien Dindo)						X								
Cystoscopy						X	X	X	X	X	X	X		
Histological confirmation of recurrence/ progression														X
Collection of FFPE tissue[3]			X											X
Urine sample collection[3]		X				X			X		X	X		X
Blood sample collection[3]		X				X			X		X	X		X

Footnotes

1. EORTC QLQ-C30 & NMIBC24, EQ-5D-3L
2. Questionnaire sent by-post directly to participant
3. If patient consented to participation in PHOTO-T (as this is archived pathology the tissue may be requested at an interval from the diagnostic resection/recurrence).

For peer review only

For peer review only - http://bmjopen.bmj.com/site/about/guidelines.xhtml

2.9. Sample size

We aim to detect an absolute reduction in recurrence at three years of 12%; from 40% (under the conservative assumption that all the patients recruited are intermediate risk patients with a probability of recurrence of 0.4 at 3 years) to 28% (similar effect sizes of photodynamic therapy are reported in both intermediate and high risk groups) this will be equivalent to a relative reduction of 30%.

Recruitment of 533 participants (214 recurrences) will detect a hazard ratio of 0.64 between experimental and control strategies and provide, using the log-rank test, 90% power at a 2-sided 5% significance level. This calculation assumes 2.5 years incremental recruitment, a minimum of three years follow-up and a 6.4% follow-up attrition at end of year three. To achieve this we plan to use 30 secondary care sites that would expect to see approximately 4,590 new bladder cancers diagnoses over 2.5 years, from which we will exclude patients with MIBC (20%) and, from the remaining NMIBCs, exclude low risk disease (50%). Furthermore, we predict only 30% of these patients will be recruited based on willingness to participate or missed opportunities for recruitment.

2.10 Health economics analysis

A within trial cost-effectiveness analysis will be conducted to calculate incremental cost per recurrence avoided and incremental cost per QALY gained over three years. Data on costs, recurrence and QALYs for each participant will be recorded in the trial and used to estimate mean cost, recurrence and QALYs for each intervention group. As the time horizon of the trial is three years these data will be discounted at 3.5% [16]. The cost, recurrence avoided and QALY data will then be

For peer review only - http://bmjopen.bmj.com/site/about/guidelines.xhtml

For peer review only

used to estimate incremental costs, recurrence avoided and QALYs and incremental cost per recurrence avoided and incremental costs per QALY.

An economic model will be developed to estimate relative rates of cost-effectiveness and cost-utility, at three years (to mirror the within trial analysis) and over a patient lifetime time horizon. An NHS perspective will be taken for the cost calculations. The model takes the form of a Markov state transition model that describes the consequences of different diagnosis and treatment strategies in terms of clinical and cost outcomes [6]. The rates of recurrence and progression recorded with the 3-year follow-up of the trial will be used to model short-term recurrence and progression rates. Further data required for the model relates to the transition and other probabilities of events beyond the 3 year follow-up, including the risk of recurrence and progression, probabilities of receiving different types of intervention should progression or recurrences occur, and risks of mortality (both from bladder cancer and other causes), will be sought through a structured systematic review of long-term outcomes of treatments of bladder cancer. The model will be used to produce estimates of costs, QALYs, recurrence rates and survival. Both costs and outcomes will be discounted at 3.5% in the base case analyses. Cost-effectiveness will be reported as incremental cost per QALY gained and incremental cost per recurrence avoided (at both 3 years and over the patient's lifetime). These data will be presented as point estimates and bootstrapping techniques will be used to estimate the statistical imprecision surrounding them. The results of this stochastic analysis will be presented as cost and QALY plots and as cost-effectiveness acceptability curves. Further deterministic sensitivity analyses will be conducted to explore other forms of uncertainty e.g. surrounding the choice of discount rate or around the unit costs of

For peer review only - http://bmjopen.bmj.com/site/about/guidelines.xhtml

equipment. The model will be probabilistic and distributions will be attached to all parameters, the shape and type of distribution will depend upon the data available and recommendations for good practice in modelling [17].

2.10. Patient and Public Involvement

Patient involvement was ensured at the early stages of protocol development and contributed to user-lead development of outcomes of value to patients in the design of the trial. Additionally, the patient journey of patient representatives was investigated through the diagnosis and treatment of bladder cancer, which includes an anonymised account impact on his quality of life. This helped understand the burden of the intervention on patients. A patient representative was involved as a co-investigator and member of the trial steering committee helping manage and analyse the implications of the research.

2.11. PHOTO-Translational side study

PHOTO Translational (PHOTO-T) aims to establish a well-characterised trial associated biorepository of longitudinal serially collected tissue samples (blood, urine and FFPE). The collection of samples from PHOTO patients is optional with every PHOTO-T consented participant collecting a urine and blood sample at baseline (pre-treatment/TURBT) and 3, 12, 24 and 36 months treatment follow-up or at recurrence (whichever comes first, predicted at 70% for the highest risk group over a trial period of 3 years). A FFPE core at baseline (plus recurrence, if occurs) will also be collected (supplement).

3. Discussion

Bladder cancer is the most frequent urothelial cancer and the overall costs for treatment and follow-up remain higher than most other cancers [18]. Achieving

For peer review only - http://bmjopen.bmj.com/site/about/guidelines.xhtml

complete resection of NMIBC with TURBT is associated with lower recurrence rates in follow-up. However, it is unclear if this translates into lower progression rates in long-term follow-up. PDD guided initial TURBT is a technology that could improve resection and ultimately reduce recurrence and the need for further treatments. Studies on PDD have demonstrated the efficacy of the technology using strict study entry requirements, for which translation into daily clinical practice is limited. Therefore, in the PHOTO trial the effectiveness of the technology as part of routine care will be demonstrated with a pragmatic clinical trial design.

PHOTO trial includes measurement of HRQoL using EQ-5D at the time of initial treatment and surveillance. The measurement of HRQoL around the time of the cystoscopy and TURBT can be particularly dynamic due to an acute deterioration in health score associated with the invasive procedure followed by a typical rapid recovery [11, 19]. Therefore, a side study was developed, where patients are recruited from the PHOTO trial to evaluate the acute deterioration in quality of life by suspected diagnosis or TURBT around the time of resection. This side study will use a time trade off exercise and the outcomes will supplement the calculation of QALYs in the health economic model.

The high costs of bladder cancer to health care systems has usually been obtained from weak data and the true costs are unclear. The pragmatic design of the PHOTO trial alongside the robust data collection for a full-health economic evaluation will provide high quality evidence of the burden of NMIBC for the NHS. Moreover, it will also provide a cost effectiveness comparison of white light vs PDD-guided initial TURBT resections.

Evidence suggests that 20 cases are required for PDD naïve surgeons to gain competency the technology. This could act as a potential confounder on the clinical

For peer review only - http://bmjopen.bmj.com/site/about/guidelines.xhtml

outcomes measured and therefore will be accounted for during analysis. Moreover, the evidence is gained from small number studies and an evaluation of the learning curve of PDD will also be carried out.

The primary outcome of the study is time to recurrence measured from the day of randomisation to the day of subsequent biopsy with pathologically proven recurrence. If decrease in time to recurrence is associated with long term patient benefits the cost-effectiveness evaluation will provide further evidence for the NHS to decide on full adoption of the technology.

For peer review only

For peer review only - http://bmjopen.bmj.com/site/about/guidelines.xhtml

4. List of abbreviations

CIS: Carcinoma in-situ

cm: centimetres

CT: Computerized tomography

DCIS: Ductal carcinoma in-site

EORTC: European Organization for Research and Treatment of Cancer

FFPE: Formalin Fixed Paraffin Embedded

HRQoL: Health related quality of life

HTA : health technology assessment

LCIS: Lobular carcinoma in situ

MIBC: Muscle invasive bladder cancer

MMC: Mitomycin-C

NHS: National Health Service

NIHR: National Institute for Health Research

NMIBC: Non-muscle invasive bladder cancer

PDD: Photo dynamic diagnosis

QALY: Quality adjusted life years

TURBT: Transurethral resection of bladder tumour

USS: Ultrasonography

WL: White light

For peer review only

Funding acknowledgement

This project was funded by the NIHR HTA (project number 11/142/02)

Department of Health disclaimer

The views expressed are those of the author(s) and not necessarily those of the NHS, the NIHR or the Department of Health.

The PHOTO trial is sponsored by Newcastle upon Tyne Hospitals NHS Foundation Trust.

Management of the study is divided between the Clinical Trials & Statistics Unit at the Institute of Cancer Research (ICR-CTSU) and Centre for Healthcare Randomised Trials (CHaRT) at the University of Aberdeen.

Independent Trial steering committee members – Mr Peter Whelan (Chair), Dr Andrew Vickers, Professor Per-Uno Malmstrom, Mr Allen Knight, Dr Dan Sjoberg.

Independent Data monitoring committee members – Dr Angela Casbard (Chair), Professor Diane Witham, Mr Robert Mills, Dr Ed Wilson, Mr Paul Silcocks.

For peer review only

For peer review only - http://bmjopen.bmj.com/site/about/guidelines.xhtml

Contributorship statement:

Zafer Tandogdu: conception and design of the work, drafting of the article, critical revision of the article and final approval of the version to be published

Rebecca Lewis: conception and design of the work, data collection, drafting of the article, critical revision of the article and final approval of the version to be published

Anne Duncan: conception and design of the work, data collection, drafting of the article, critical revision of the article and final approval of the version to be published

Alison McDonald: conception and design of the work, data collection, drafting of the article, critical revision of the article and final approval of the version to be published

Luke Vale: conception and design of the work, critical revision of the article and final approval of the version to be published

Steven Penegar: data collection, critical revision of the article and final approval of the version to be published

Jing Shen: design of the work, critical revision of the article and final approval of the version to be published

PHOTO TMG members: Trial management, data collection and final approval of the version to be published

Graeme Maclennan: design of the work, critical revision of the article and final approval of the version to be published

John Norrie: conception and design of the work, critical revision of the article and final approval of the version to be published

Emma Hall: conception and design of the work, critical revision of the article and final approval of the version to be published

Rakesh Heer: conception and design of the work, drafting of the article, critical revision of the article and final approval of the version to be published

For peer review only - http://bmjopen.bmj.com/site/about/guidelines.xhtml

For peer review only

For peer review only - http://bmjopen.bmj.com/site/about/guidelines.xhtml

5. References

1. UK, C.R. *Bladder cancer statistics*. Cancer statistics 2013 [cited 2016 05-09-2016]; Available from: http://www.cancerresearchuk.org/health-professional/cancer-statistics/statistics-by-cancer-type/bladder-cancer.
2. Epstein, J.I., et al., *The World Health Organization/International Society of Urological Pathology consensus classification of urothelial (transitional cell) neoplasms of the urinary bladder. Bladder Consensus Conference Committee.* Am J Surg Pathol, 1998. **22**(12): p. 1435-48.
3. Brausi, M., et al., *Variability in the recurrence rate at first follow-up cystoscopy after TUR in stage Ta T1 transitional cell carcinoma of the bladder: a combined analysis of seven EORTC studies.* Eur Urol, 2002. **41**(5): p. 523-31.
4. Bryan, R.T., et al., *Mechanisms of recurrence of Ta/T1 bladder cancer.* Ann R Coll Surg Engl, 2010. **92**(6): p. 519-24.
5. Babjuk, M., et al., *EAU Guidelines on Non-Muscle-invasive Urothelial Carcinoma of the Bladder: Update 2016.* Eur Urol, 2017. **71**(3): p. 447-461.
6. Mowatt, G., et al., *Photodynamic diagnosis of bladder cancer compared with white light cystoscopy: Systematic review and meta-analysis.* Int J Technol Assess Health Care, 2011. **27**(1): p. 3-10.
7. Sylvester, R.J., et al., *Predicting recurrence and progression in individual patients with stage Ta T1 bladder cancer using EORTC risk tables: a combined analysis of 2596 patients from seven EORTC trials.* Eur Urol, 2006. **49**(3): p. 466-5; discussion 475-7.
8. Rink, M., et al., *Hexyl Aminolevulinate-Guided Fluorescence Cystoscopy in the Diagnosis and Follow-up of Patients with Non-Muscle-invasive Bladder Cancer: A Critical Review of the Current Literature.* European urology, 2013. **64**(4): p. 624-38.
9. Sangar, V.K., et al., *The economic consequences of prostate and bladder cancer in the UK.* BJU Int, 2005. **95**(1): p. 59-63.
10. Leal, J., et al., *Economic Burden of Bladder Cancer Across the European Union.* Eur Urol, 2016. **69**(3): p. 438-47.
11. Yoshimura, K., et al., *Impact of superficial bladder cancer and transurethral resection on general health-related quality of life: an SF-36 survey.* Urology, 2005. **65**(2): p. 290-4.
12. Porter, M.P. and D.F. Penson, *Health related quality of life after radical cystectomy and urinary diversion for bladder cancer: a systematic review and critical analysis of the literature.* The Journal of urology, 2005. **173**(4): p. 1318-22.
13. *Comprehensive molecular characterization of urothelial bladder carcinoma.* Nature, 2014. **507**(7492): p. 315-22.
14. Miremami, J. and N. Kyprianou, *The promise of novel molecular markers in bladder cancer.* Int J Mol Sci, 2014. **15**(12): p. 23897-908.
15. Botteman, M.F., et al., *Quality of life aspects of bladder cancer: a review of the literature.* Qual Life Res, 2003. **12**(6): p. 675-88.
16. Miners, A., *Estimating 'costs' for cost-effectiveness analysis.* Pharmacoeconomics, 2008. **26**(9): p. 745-51.
17. Kaltenthaler, E., et al., *NICE Decision Support Unit Technical Support Documents*, in *NICE DSU Technical Support Document 13: Identifying and Reviewing Evidence to Inform the Conceptualisation and Population of Cost-Effectiveness Models*. 2011, National Institute for Health and Care Excellence (NICE)

unless otherwise stated. All rights reserved.: London.
18. Johnson, D.C., P.S. Greene, and M.E. Nielsen, *Surgical advances in bladder cancer: at what cost?* Urol Clin North Am, 2015. **42**(2): p. 235-52, ix.

For peer review only - http://bmjopen.bmj.com/site/about/guidelines.xhtml

19. van der Aa, M.N., et al., *Patients' perceived burden of cystoscopic and urinary surveillance of bladder cancer: a randomized comparison.* BJU Int, 2008. **101**(9): p. 1106-10.

6. Figure Legends:

Figure 1: Tumour, Node, Metastasis (TNM) system of bladder cancer [2]

Figure 2: PHOTO trial study design summary

For peer review only

For peer review only - http://bmjopen.bmj.com/site/about/guidelines.xhtml

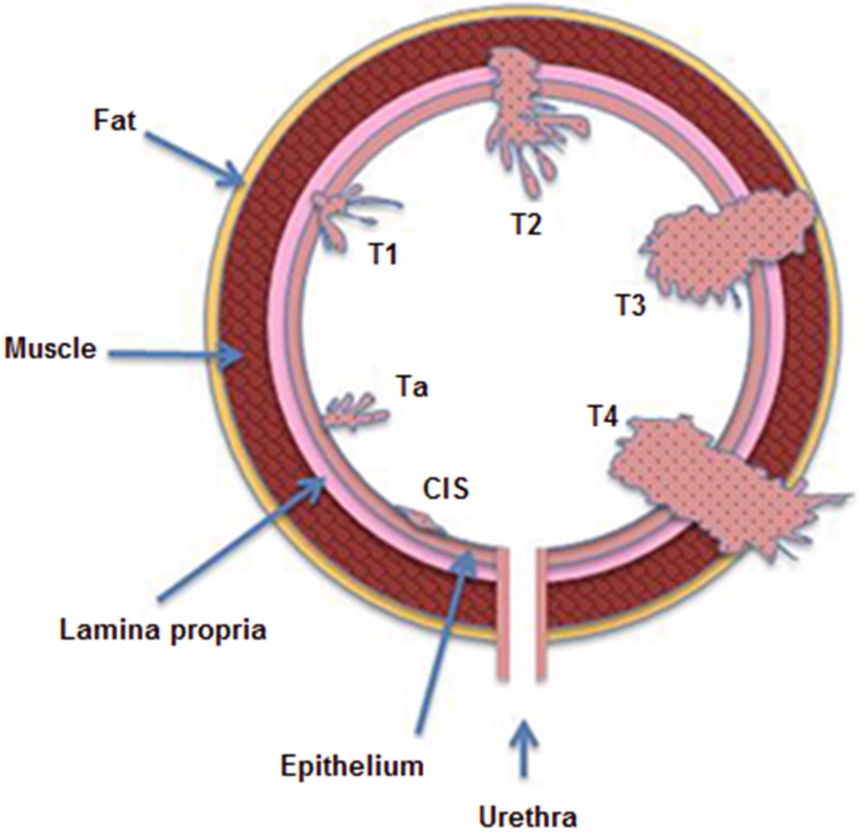

PHOTO Trial study design summary.

90x90mm (300 x 300 DPI)

APPENDIX-I: NICR PHOTO-T study

Sample Collection (per participant):

(a) 2 x blood samples totalling 12.5ml (10ml Streck blood collection tube for circulating DNA analysis and 2.5ml PAXgene blood collection tube for circulating RNA analysis); (b) 2 x urine samples totalling 200ml (1 x 100ml for immediate translational processing and 1 x 100ml for biorepository storage), (c) 1 x FFPE core at baseline (plus core from recurrence, if occurs).

In total, 20 serially collected samples will be collected per PHOTO-T consented participant over the trial period (36 months), comprising 10 urine, 10 blood (5 DNA and 5 RNA) and 1 FFPE tumour tissue block. Collected at baseline (pre-treatment/TURBT) and treatment follow-up at 3, 12, 24 and 36 months or at recurrence (whichever comes first, predicted at 70% for the highest risk group over a trial period of 3 years).

Serial blood samples are requested for collection in clinic by research staff at participating investigator sites. Urine samples are provided at home by participants using specialist ‘home collection’ kits (home collection is preferable as sample quality in terms of genomic output is superior to clinically collected specimens) and FFPE blocks requested from Histopathology Departments of participating investigator sites retrospectively at the end of the trial.

For peer review only

BMJ Open

PHOTOdynamic versus white light-guided treatment of non-muscle invasive bladder cancer: A randomised trial of clinical and cost effectiveness

Journal:	*BMJ Open*
Manuscript ID	bmjopen-2018-022268.R1
Article Type:	Protocol
Date Submitted by the Author:	03-May-2019

Complete List of Authors:	tandogdu, zafer; Northern Institute for Cancer Research<br>Lewis, Rebecca; Institute of Cancer Research<br>Duncan, Anne; University of Aberdeen, Centre for Healthcare Randomised Trials (CHaRT)<br>McDonald, Alison; University of Aberdeen, Centre for Healthcare Randomised Trials (CHaRT)<br>Vale, Luke; Newcastle University, Health Economics Group, Institute of Health and Society<br>Penegar, Steven; Institute of Cancer Research<br>Shen, Jing; Institute of Health & Society<br>Kelly, John; University College London Hospitals NHS Foundation Trust, Urology<br>Pickard, Robert; Newcastle University Institute of Cellular Medicine<br>NDow, James; University of Aberdeen, Surgery<br>Ramsay, Craig; Health Service research unit, University of Aberdeen<br>Mostafid, Hugh; Hampshire Hospitals NHS Foundation Trust<br>Mariappan, Paramananthan; Western General Hospital, urology<br>Nabi, Ghulam; University of Dundee, Medicine<br>creswell, Joanne; South Tees Hospitals NHS Foundation Trust<br>Lazarowicz, Henry; Royal Liverpool and Broadgreen University Hospitals NHS Trust<br>McGrath, John; Royal Devon and Exeter NHS Foundation Trust, Urology<br>Taylor, Ernest; PHOTO Trial TMG<br>Clark, Emma; Northern Institute for Cancer Research<br>Maclennan, Graeme; University of Aberdeen, Centre for Healthcare Randomised Trials (CHaRT)<br>Norrie, John; Edinburgh Clinical Trials Unit, University of Edinburgh No. 9, Bioquarter<br>Hall, Emma; The Institute of Cancer Research<br>Heer, Rakesh; Northern Institute for Cancer Research

**Primary Subject Heading**:	Urology
Secondary Subject Heading:	Health economics, Oncology
Keywords:	bladder cancer, photodynamic diagnosis, quality of life, HEALTH ECONOMICS, Cost effectiveness, Sample biorepository

SCHOLARONE™
Manuscripts

Title page

PHOTOdynamic versus white light-guided treatment of non-muscle invasive bladder cancer: A randomised trial of clinical and cost effectiveness

Authors: Zafer Tandogdu[1](*), Rebecca Lewis[2](*), Anne Duncan[3](*), Alison McDonald[3], Luke Vale[4], Steven Penegar[2], Jing Shen[4], John D. Kelly[5](₸), Robert Pickard[6], James NDow[7](₸), Craig Ramsay[8](₸), Hugh Mostafid[9](₸), Paramananthan Mariappan [10](₸), Ghulam Nabi[11](₸), Joanne Creswell[12](₸), Henry Lazarowicz[13](₸), John McGrath[14](₸), Ernest Taylor[15](₸), Emma Clark[1](₸), Graeme Maclennan[3], John Norrie[16](**), Emma Hall[2](**), Rakesh Heer[1](**)

*Authors have provided equal contribution and are joint first authors.

**Joint senior authors.

₸ PHOTO Trial Management Group – to be listed as grouped authors.

1. Northern Institute for Cancer Research, Newcastle University, Newcastle upon tyne, UK
2. The Institute of Cancer Research, London, UK
3. Centre for Healthcare Randomised Trials (CHaRT), University of Aberdeen, Aberdeen, UK
4. Institute for Health and Society, Newcastle University, Newcastle upon Tyne, UK

For peer review only - http://bmjopen.bmj.com/site/about/guidelines.xhtml

5. University College London Hospitals NHS Foundation Trust, Urology Department, London, UK
6. Institute of Cellular Medicine, Newcastle University, UK
7. Academic Urology Unit, University of Aberdeen, Aberdeen, UK
8. Health Service Research Unit, University of Aberdeen, Aberdeen, UK
9. Basingstoke and North Hampshire NHS Foundation Trust, UK
10. Western General Hospital, Urology, Edinburgh, UK
11. University of Dundee, Medicine, Dundee, UK
12. South Tees Hospitals NHS Trust ,Middlesbrough, UK
13. Royal Liverpool and Broadgreen university Hospitals NHS Trust, UK
14. Royal Devon and Exeter Hospital NHS Trust, UK
15. PHOTO Trial patient representative, UK
16. Edinburgh Clinical Trials Unit, Edinburgh University, Edinburgh, UK

Corresponding authors:

1. Graeme Maclennan:
    - e-mail: G.maclennan@abdn.ac.uk
    - Phone:01224 438147

Key words:

- Bladder cancer
- Photodynamic diagnosis
- Quality of life
- Health economics
- Cost effectiveness

For peer review only

- Sample biorepository

The authors declare that they have no competing interests.

For peer review only

For peer review only - http://bmjopen.bmj.com/site/about/guidelines.xhtml

Abstract

Introduction: Bladder cancer is the most frequently occurring tumour of the urinary system. Ta, T1 tumours and carcinoma in situ (CIS) are grouped as non-muscle invasive bladder cancer (NMIBC), which can be effectively treated by transurethral resection of bladder tumour (TURBT). There are limitations to the visualisation of tumours with conventional TURBT using white light illumination within the bladder. Incomplete resections occur from the failure to identify satellite lesions or the full extent of the tumour leading to recurrence and potential risk of disease progression. To improve complete resection, photodynamic diagnosis (PDD) has been proposed as a method that can enhance tumour detection and guide resection. The objective of the current research is to determine whether PDD-guided TURBT is better than conventional white light surgery and whether it is cost-effective.

Methods and Analysis: PHOTO is a pragmatic multi-centre randomised controlled trial (open parallel group, non-masked, superiority trial) comparing the intervention of PDD-guided TURBT with standard white light resection in newly diagnosed intermediate and high risk NMIBC within the UK NHS setting. Clinical effectiveness is measured with time to recurrence. Cost-effectiveness is assessed within trial via the calculation of incremental cost per recurrence avoided and incremental cost per quality adjusted life per year (QALY) gained over three years, and over long term through a modelling exercise over patients' life time.

Ethics and dissemination: Formal ethics review was undertaken with a favourable opinion, in line with UK regulatory procedures (REC reference number: 14/NE/1062; Trial registration: ISRCTN84013636). If reductions in time to recurrence is associated with long term patient benefits the cost-effectiveness evaluation will provide further evidence to inform adoption of the technology. Findings will be

For peer review only - http://bmjopen.bmj.com/site/about/guidelines.xhtml

shared in lay media such as patient and charity forums and will be presented at key meetings and published in academic literature.

Article Summary:

Strengths and limitations of the PHOTO study include;

- the effectiveness of the photodynamic diagnosis (PDD) for the initial resection of intermediate and high risk non-muscle invasive bladder cancer as part of routine care will be demonstrated with a pragmatic clinical trial design,
- full-health economic evaluation will provide high quality evidence of the burden of NMIBC for the NHS
- a well-characterised trial associated biorepository of longitudinal serially collected tissue samples

For peer review only

For peer review only - http://bmjopen.bmj.com/site/about/guidelines.xhtml

1. Background

Bladder cancer is the most frequently occurring tumour of the urinary system [1]. Staging of bladder cancer is described using the Tumour, Node, Metastasis (TNM) system [2]. Tumours confined to the epithelial lining (urothelium) are classified as stage Ta and those invading the lamina propria are classified as stage T1.

Ta and T1 tumours can be removed by transurethral resection (TURBT) that involves passing a cystoscope through the urethra into the bladder and resecting the tumour under direct visualisation. For therapeutic purposes Ta and T1 tumours are grouped together as non-muscle invasive bladder cancer (NMIBC). Grade (microscopic characteristics of the tumour cells) can be used to describe aggressiveness of cancers and are characterised as either low grade (relatively benign) or high grade (aggressive). NMIBC also include flat, high-grade tumours that are confined to the epithelium classified as carcinoma in situ (CIS).

Complete resection of the tumour with TURBT is essential to obtain good prognosis. It is thought that failure to identify satellite tumours or to appreciate the full extent of the tumours visualised during resection using conventional white light cystoscopy may be a factor in 20-40% of recurrent bladder tumours [3, 4]. Incomplete resection with TURBT is also associated with staging errors. In order to correct the staging errors associated with initial TURBT a second resection within 2-6 weeks is suggested for select group patients [5]. It has been postulated that development in cystoscopy imaging can improve resections and decrease the need for a second resection [6].

Recurrence and stage progression to muscle invasive (T2-T4) or metastatic cancer is more likely to occur in those with high-grade tumours with concomitant

For peer review only - http://bmjopen.bmj.com/site/about/guidelines.xhtml

CIS. CIS in particular can be easily missed using conventional white light guided resection [6].

Surveillance of NMIBC is carried out with cystoscopy to detect recurrence early and allow treatment before progression. Clinical guidelines tailor follow up protocols according to the risk groups (low, intermediate and high) developed using clinical and histological parameters [7]. Advised follow-up of low risk is at three months and if negative the next cystoscopy is scheduled for nine months later and then yearly for five years. Patients with high-risk tumours have cystoscopy and urine cytology at three months. If negative, it is repeated every three months for two years, then every six months until five years, and annually thereafter [5]. The intensity of cystoscopic follow up for patients with intermediate risk is not clearly defined, for which a follow-up scheme in-between those described for low and high risk and is adapted according to personal and subjective factors [5].

1.1. Photodynamic diagnosis of NMIBC:

As an attempt to improve resection rates, photodynamic diagnosis (PDD) has been developed to enhance tumour detection and guide resection. A cystoscopy image of WL vs PDD is presented in figure 1. Meta-analyses and systematic reviews of PDD guided treatment of NMIBC have shown efficacy in tumour detection and reduction in residual tumour compared with white light cystoscopy alone. These findings translate into reduced recurrence rates [6, 8]. However, these trials were efficacy studies and the systematic review called for a pragmatic study to allow better interpretation of possible benefit into daily clinical practice.

1.2. Health economics of NMIBC:

For peer review only - http://bmjopen.bmj.com/site/about/guidelines.xhtml

For peer review only

NMIBC is one of the most costly cancers to manage on a per patient basis because of its high prevalence, high recurrence rate, need for adjuvant treatments and the requirement for long-term cystoscopic surveillance. The total cost of treatment and 5-year follow-up of patients with NMIBC diagnosed in the United Kingdom has increased from £73 million to £213 million from 2001 to 2012 (inflation corrected) [9, 10]. From a patient perspective, there often are considerable anxieties about recurrences, transurethral resection and progression, requiring additional therapies with potential mortality and long-term morbidity (e.g. radical surgery). Transurethral resection itself is associated with reduced quality of life, including both mental and physical health domains; although these effects are usually transient [11]. Substantial effects on health related quality of life (HRQoL) are most likely to come from adjuvant intravesical treatments and radical or palliative treatments for progression [12]. The cost-effectiveness of NMIBC treatment strategies has not been widely studied.

1.3. NMIBC biomarkers and clinical impact:

To date, existing non-invasive commercial biomarkers (primarily urinary) are not embedded in routine clinical practice due to poor sensitivity, specificity and lack of evidence. Several research bodies have recognized the lack of clinically useful biomarkers for bladder cancer. “Fit for purpose” sample resources accessible to high-throughput ‘omic’ technologies will afford the greatest opportunity to generate translational hypotheses and ensure clinical validity and utility of putative candidate markers/signatures [13, 14]. Robust, ‘future-proof’, longitudinal serial sample archives providing critical insights of the natural history of bladder cancer correlated with clinical detail for retrospective translational biomarker discovery, are lacking.

For peer review only

1.4. Current research objectives:

More efficient management strategies to reduce NMIBC recurrence and hence decrease both the burden to patients and costs are urgently needed. PDD-guided initial TURBT has been identified as a technique that can help achieve these aims. The objective of the current research (PHOTO trial) is to determine whether photodynamic surgery guided by a fluorescent tumour marker is better than conventional white light surgery in the cystoscopic treatment of people with intermediate and high risk cancers confined to the bladder lining and whether its implementation is cost-effective. The trial includes a full assessment of the costs of patient management through the care pathway. Individual patient data from this trial will be used for subsequent mathematical modelling studies to investigate safe monitoring frequency. The Photodynamic versus white light-guided treatment of non-muscle invasive bladder cancer trial has the following research objectives:

i. Primary objectives:

Clinical effectiveness: To compare time to recurrence for each of the two treatment strategies, with a principal point of interest at 3 years.

Cost-effectiveness: To evaluate cost-effectiveness as measured by the incremental cost per recurrence avoided and the cost-utility as measured by the incremental cost per quality-adjusted life year (QALY) gained at three years and over patients' life time.

ii. Secondary objectives:

a. To measure relative rates of disease progression at three years.
b. To measure relative harms and safety.
c. Patient lifetime HRQoL and cancer-specific survival.

For peer review only - http://bmjopen.bmj.com/site/about/guidelines.xhtml

iii. Additional objectives:

a. To model the safest and most cost-effective cystoscopic follow-up surveillance schedule;

b. To evaluate the learning curve for the procedure and account for its effects on outcomes of both PDD-guided and standard white light resections;

c. To establish a well-characterised cohort of serial samples from patients with intermediate and high-risk NMIBC including clinical data, urine, blood and tumour specimens for separately funded translational research.

2. Methods & Design

2.1. Study design

PHOTO is a multi-centre randomised open parallel group non-masked superiority trial comparing the intervention of PDD guided bladder tumour resection with standard white light resection in patients with newly diagnosed intermediate and high risk NMIBC. Apart from initial treatment, both groups will receive standard care, including single dose intravesical mitomycin C within 24 hours of initial resection, surveillance according to risk-adjusted schedules and adjuvant therapy as indicated by current practice guidelines. The target number of patients to be recruited is 533 with a trial specific follow-up of at least 36 months for each individual. The outline of the study protocol is shown in Figure 2.

For peer review only - http://bmjopen.bmj.com/site/about/guidelines.xhtml

For peer review only

2.2. Intervention

The interventions being compared within PHOTO trial are:

i. Photo dynamic diagnosis (PDD) guided trans-urethral resection (TURBT) (experimental group) vs;

ii. Standard white light TURBT (control group)

All participants, unless there are clinical contra-indications, receive intravesical mitomycin C (40 mg in 40 ml saline), ideally within 6 hours following TURBT but otherwise within the inpatient setting before discharge.

2.2.1. Technique of photodynamic diagnosis

PDD requires preliminary instillation of the photosensitiser hexaminolevulinate (85 mg in 50 ml of phosphate buffered saline) into the participant's bladder through a urethral catheter. Participants are asked not to pass urine for at least one hour after insertion. Following operating theatre preparation according to local standard procedures and under appropriate anaesthesia, participants undergo TURBT of their bladder tumour under blue light (wavelength 380-450 nm) illumination of the bladder. The equipment required includes a specialised light source, cystoscope, light cables and cameras. When using PDD, normal bladder epithelium appears blue whilst red areas should be considered suspicious and should be resected.

2.2.2. Technique of standard white light cystoscopy

The control group does not have any preliminary photosensitiser instillation and undergo standard tumour localisation and resection under white light (wavelength 400-800 nm) illumination of the bladder.

For peer review only - http://bmjopen.bmj.com/site/about/guidelines.xhtml

2.3. Participants

In the PHOTO trial adult patients with a suspected new diagnosis of intermediate or high risk NMIBC are studied. Participants are identified prior to initial resection based on results of preliminary visual assessment via cystoscopy or imaging performed as part of standard evaluation for suspected urinary tract malignancy. Patients with the following criteria are included in the PHOTO trial:

- Adult men and women aged ≥ 16 years.
- First suspected diagnosis of bladder cancer.
- Visual/ultrasound/Computerized tomography (CT) diagnosis of intermediate/high risk NMIBC.
- White light visual appearances of intermediate or high risk disease (≥3cm OR two or more tumours OR flat velvety erythematous changes alerting a clinical suspicion of CIS).

  OR

  Suspicion of papillary bladder tumour ≥3cm based on ultrasound or CT scanning (without hydronephrosis).
- Written informed consent for participation prior to any study specific procedures.
- Willing to comply with the following life style guidelines:
  - Female participants must be surgically sterile or be post-menopausal, or must agree to use effective contraception after joining the study and for 7 days after treatment. Female participants must not breast feed for 7 days after treatment.

For peer review only - http://bmjopen.bmj.com/site/about/guidelines.xhtml

- Male participants must be surgically sterile or must agree to use effective contraception after joining the study and for 7 days after treatment.
- Effective contraception is defined as two forms of contraception, including one barrier method.

Exclusion criteria applied in the PHOTO trial are:

- Visual evidence of low risk NMIBC (solitary tumour < 3cm).
- Visual evidence of MIBC on preliminary cystoscopy, i.e. non-papillary or sessile mass (attached directly by its base without a stalk).
- Imaging evidence of MIBC – CT/USS (this includes the presence of hydronephrosis, which may be present despite clear imaging of MIBC in the bladder).
- Upper tract (kidney or ureteric) tumours on imaging.
- Any other malignancy in the past 2 years (except: non-melanomatous skin cancer cured by excision, adequately treated carcinoma in situ of the cervix, DCIS/LCIS of the breast or prostate cancer in patients who have a life expectancy of >5 years upon trial entry).
- Evidence of metastases.
- Porphyria or known hypersensitivity to porphyrins.
- Known pregnancy (based on history and without formal testing, in keeping with day-to-day NHS practice of PDD use).
- Any other conditions that in the Principal Investigator's opinion would contraindicate protocol treatment.
- Unable to provide informed consent.

For peer review only - http://bmjopen.bmj.com/site/about/guidelines.xhtml

- Unable or unwilling to complete follow-up schedule (including HRQoL questionnaires).

2.4. Informed consent-ethics approval

Favourable ethical opinion for this research was provided by the Newcastle & North Tyneside 2 ethics committee (REC reference number: 14/NE/1062) in July 2014. The study complies with the Helsinki Declaration and the principles of Good Clinical Practice (GCP).

Potential participants are identified mainly through rapid access haematuria clinics at participating sites. An eligibility checklist is completed by the local Principal Investigator (or delegate) to assess fulfilment of the entry criteria for all patients considered for the study. Information from the diagnostic cystoscopy is used to assess eligibility.

All potentially eligible patients are provided with an information sheet to explain why they have been approached and the nature of the study. Eligible patients are asked to provide written informed consent for the study only after they have had sufficient time to consider the trial and had the opportunity to have any further questions addressed.

For peer review only

2.5. Recruitment and randomisation

Eligible patients are centrally randomised using either the secure web-based or the 24-hour Interactive Voice Response randomisation system at the Centre for Healthcare Randomised Trials (CHaRT) in Aberdeen, using minimisation by centre and gender, to allocate participants 1:1 to the control and experimental groups. The minimisation algorithm incorporates a random element in order to prevent

For peer review only - http://bmjopen.bmj.com/site/about/guidelines.xhtml

deterministic treatment allocation. The trial was opened on the 23rd of October 2014 and recruitment was completed on 14th of February 2018. Over this period 22 centres joined the study and contributed to recruitment.

2.6. Outcome measures

2.6.1. Primary outcome measures

Clinical effectiveness: Time to recurrence is measured as time from randomisation to first recurrence.

Cost effectiveness: A health economic model will be developed to calculate incremental cost per recurrence avoided and incremental cost per QALY gained over three years.

2.6.2. Secondary outcome measures

Clinical effectiveness:

- Adverse events and complications up to 3 months from initial or second TURBT are captured and will be included in analysis.
- HRQOL is captured for each participant at baseline (prior to knowledge of treatment allocation), following surgery and at 3, 6, 12, 18, 24 and 36 months after randomisation.
- Disease progression is captured within the trial time. Patient life time projections will be made by using the trial data at baseline and supplementing as necessary from other published data.

For peer review only - http://bmjopen.bmj.com/site/about/guidelines.xhtml

For peer review only

- Overall survival and bladder cancer specific survival will be compared between the two treatment arms. Minimum follow-up of the last included patient will be 3 years and maximum expected follow-up is 66 months.

Cost effectiveness:

- Estimation of the incremental cost per recurrence avoided using the economic model over the patients' lifetime.
- Estimation of the incremental cost per QALY gained using the economic model over the patients' lifetime.

2.6.3. Additional outcomes measures

Schedules for follow-up: Using data from within the trial and, if appropriate, from other relevant sources, the risk of recurrence at each interval surveillance cystoscopy will be described to then model the most safe and efficient surveillance follow-up schedule.

The effect of PDD guided resection experience (learning curve) on clinical effectiveness: A subgroup analysis comparing outcomes from PDD-experienced and PDD-naïve surgeons (determined at baseline) will be conducted. Also for PDD-naïve surgeons an assessment of learning curve will be undertaken by comparing increasing experience and recurrence, in both PDD and WL resections.

2.7. Tracking and monitoring adverse events

Direct surgically related post-operative events occurring within 30 days following the first TURBT or second TURBT if required will be assessed using The Clavien Dindo classification for surgical complications. Events occurring up to 3 months after TURBT (second TURBT if required) will be assessed and recorded using the

For peer review only - http://bmjopen.bmj.com/site/about/guidelines.xhtml

For peer review only

National Cancer Institute Common Terminology Criteria for Adverse Events (CTCAE) v4.0 framework (http://ctep.cancer.gov/) [15].

2.8. Trial assessment and measures

PHOTO trial schedule of assessment and investigations are summarised in Table 1. Routine attendances for diagnosis and staging of new bladder cancers are used to establish eligibility, which includes obtaining the medical history. Eligible patients who consent for the trial are administered HRQoL questionnaires prior to primary TURBT and prior to discharge.

Time to recurrence will be measured from the day of randomisation to the day of subsequent biopsy with pathologically proven recurrence. Data will be collected from the following routine visits; 3, 6, 9, 12, 18, 24 and 36 months post initial TURBT (or second TURBT if required). Associated costs and changes in HRQoL will be measured. These will be collected by postal questionnaires sent directly to participants at 3, 6, 12, 18, 24 & 36 months post randomisation.

Disease progression will be assessed using results of further resection or imaging during follow-up. Progression will be defined as increase of stage into MIBC or development of nodal or metastatic disease. In addition, patients showing failure to respond to intravesical treatment (e.g. BCG failure) will also be captured.

The relative changes in HRQoL resulting from the physical and psychological benefit together with any harms associated with each strategy and with subsequent necessary cancer treatment will be measured using the generic EQ-5D-3L questionnaire, cancer specific EORTC-QLQ-C30 and disease-specific EORTC QLQ-NMIBC24 questionnaire completed by the participant.

For peer review only - http://bmjopen.bmj.com/site/about/guidelines.xhtml

Effect of PDD guided resection experience on clinical effectiveness: All recruiting surgeons will complete a learning curve questionnaire to elicit their white light and PDD resection experience prior to any recruitment. The subsequent accruing experience of each surgeon will be captured on case report forms. Early recurrence (12 weeks) will be used as a proxy of incomplete resection.

Table 1, Schedule of investigations/assessments in PHOTO trial

Visit/Assessment	Pre- randomisation screening	Pre-treatment	TURBT	Prior to discharge	Surveillance									At first disease recurrence/progression
					Second TURBT (as clinically indicated)	3 months post treatment	6 months post treatment	9 months post treatment	12 months post treatment	18 months post treatment	24 months post treatment	36 months post treatment	Annually thereafter	
Visual diagnosis of IR/HR NMIBC	X												According to EAU guidelines	Treatment according to local practice
Medical history	X													
HRQoL questionnaire[1]		X		X	X	X[2]	X[2]	X[2]	X[2]	X[2]	X[2]	X[2]		
TURBT according to treatment allocation with post treatment MMC instillation			X											
Second TURBT, if required, according to treatment allocation					X									
Assessment of adverse events (CTCAE & Clavien Dindo)						X								
Cystoscopy						X	X	X	X	X	X	X		
Histological confirmation of recurrence/ progression														X
Collection of FFPE tissue[3]			X											X
Urine sample collection[3]		X				X			X		X	X		X
Blood sample collection[3]		X				X			X		X	X		X

Footnotes

For peer review only

For peer review only - http://bmjopen.bmj.com/site/about/guidelines.xhtml

1. EORTC QLQ-C30 & NMIBC24, EQ-5D-3L
2. Questionnaire sent by-post directly to participant
3. If patient consented to participation in PHOTO-T (as this is archived pathology the tissue may be requested at an interval from the diagnostic resection/recurrence).

For peer review only

For peer review only - http://bmjopen.bmj.com/site/about/guidelines.xhtml

2.9. Sample size

We aim to detect an absolute reduction in recurrence at three years of 12%; from 40% (under the conservative assumption that all the patients recruited are intermediate risk patients with a probability of recurrence of 0.4 at 3 years) to 28% (similar effect sizes of photodynamic therapy are reported in both intermediate and high risk groups) this will be equivalent to a relative reduction of 30%.

Recruitment of 533 participants (214 recurrences) will detect a hazard ratio of 0.64 between experimental and control strategies and provide, using the log-rank test, 90% power at a 2-sided 5% significance level. This calculation assumes 2.5 years incremental recruitment, a minimum of three years follow-up and a 6.4% follow-up attrition at end of year three. To achieve this we plan to use 30 secondary care sites that would see new bladder cancers diagnoses, from which we will exclude patients with MIBC (20%) and, from the remaining NMIBCs, exclude low risk disease (50%). Furthermore, we predict only 30% of these patients will be recruited based on willingness to participate or missed opportunities for recruitment.

2.10 Health economics analysis

A within trial cost-effectiveness analysis will be conducted to calculate incremental cost per recurrence avoided and incremental cost per QALY gained over three years. Data on costs, recurrence and QALYs for each participant will be recorded in the trial and used to estimate mean cost, recurrence and QALYs for each intervention group. As the time horizon of the trial is three years these data will be discounted at 3.5% [16]. The cost, recurrence avoided and QALY data will then be used to estimate incremental costs, recurrence avoided and QALYs and incremental cost per recurrence avoided and incremental costs per QALY.

For peer review only - http://bmjopen.bmj.com/site/about/guidelines.xhtml

An economic model will be developed to estimate relative rates of cost-effectiveness and cost-utility, at three years (to mirror the within trial analysis) and over a patient lifetime time horizon. An NHS perspective will be taken for the cost calculations. The model takes the form of a Markov state transition model that describes the consequences of different diagnosis and treatment strategies in terms of clinical and cost outcomes [6]. The rates of recurrence and progression recorded with the 3-year follow-up of the trial will be used to model short-term recurrence and progression rates. Further data required for the model relates to the transition and other probabilities of events beyond the 3 year follow-up, including the risk of recurrence and progression, probabilities of receiving different types of intervention should progression or recurrences occur, and risks of mortality (both from bladder cancer and other causes), will be sought through a structured systematic review of long-term outcomes of treatments of bladder cancer. The model will be used to produce estimates of costs, QALYs, recurrence rates and survival. Both costs and outcomes will be discounted at 3.5% in the base case analyses. Cost-effectiveness will be reported as incremental cost per QALY gained and incremental cost per recurrence avoided (at both 3 years and over the patient's lifetime). These data will be presented as point estimates and bootstrapping techniques will be used to estimate the statistical imprecision surrounding them. The results of this stochastic analysis will be presented as cost and QALY plots and as cost-effectiveness acceptability curves. Further deterministic sensitivity analyses will be conducted to explore other forms of uncertainty e.g. surrounding the choice of discount rate or around the unit costs of equipment. The model will be probabilistic and distributions will be attached to all

For peer review only - http://bmjopen.bmj.com/site/about/guidelines.xhtml

parameters, the shape and type of distribution will depend upon the data available and recommendations for good practice in modelling [17].

2.10. Patient and Public Involvement

Patient involvement was ensured at the early stages of protocol development and contributed to user-lead development of outcomes of value to patients in the design of the trial. Additionally, the patient journey of patient representatives was investigated through the diagnosis and treatment of bladder cancer, which includes an anonymised account impact on his quality of life. This helped understand the burden of the intervention on patients. A patient representative was involved as a co-investigator and member of the trial steering committee helping manage and analyse the implications of the research.

2.11. PHOTO-Translational side study

PHOTO Translational (PHOTO-T) aims to establish a well-characterised trial associated biorepository of longitudinal serially collected tissue samples (blood, urine and FFPE). The collection of samples from PHOTO patients is optional with every PHOTO-T consented participant collecting a urine and blood sample at baseline (pre-treatment/TURBT) and 3, 12, 24 and 36 months treatment follow-up or at recurrence (whichever comes first, predicted at 70% for the highest risk group over a trial period of 3 years). A FFPE core at baseline (plus recurrence, if occurs) will also be collected (supplement).

3. Discussion

Bladder cancer is the most frequent urothelial cancer and the overall costs for treatment and follow-up remain higher than most other cancers [18]. Achieving complete resection of NMIBC with TURBT is associated with lower recurrence rates

For peer review only - http://bmjopen.bmj.com/site/about/guidelines.xhtml

in follow-up. However, it is unclear if this translates into lower progression rates in long-term follow-up. PDD guided initial TURBT is a technology that could improve resection and ultimately reduce recurrence and the need for further treatments. Studies on PDD have demonstrated the efficacy of the technology using strict study entry requirements, for which translation into daily clinical practice is limited. Therefore, in the PHOTO trial the effectiveness of the technology as part of routine care will be demonstrated with a pragmatic clinical trial design.

PHOTO trial includes measurement of HRQoL using EQ-5D at the time of initial treatment and surveillance. The measurement of HRQoL around the time of the cystoscopy and TURBT can be particularly dynamic due to an acute deterioration in health score associated with the invasive procedure followed by a typical rapid recovery [11, 19]. Therefore, a side study was developed, where patients are recruited from the PHOTO trial to evaluate the acute deterioration in quality of life by suspected diagnosis or TURBT around the time of resection. This side study will use a time trade off exercise and the outcomes will supplement the calculation of QALYs in the health economic model.

The high costs of bladder cancer to health care systems has usually been obtained from weak data and the true costs are unclear. The pragmatic design of the PHOTO trial alongside the robust data collection for a full-health economic evaluation will provide high quality evidence of the burden of NMIBC for the NHS. Moreover, it will also provide a cost effectiveness comparison of white light vs PDD-guided initial TURBT resections.

Evidence on the required cases for PDD naïve surgeons to gain competency the technology is weak. This could act as a potential confounder on the clinical outcomes measured and therefore will be accounted for during analysis. Moreover, an

For peer review only - http://bmjopen.bmj.com/site/about/guidelines.xhtml

evaluation of the learning curve of PDD will also be carried out using the forms filled in by surgeons.

The primary outcome of the study is time to recurrence measured from the day of randomisation to the day of subsequent biopsy with pathologically proven recurrence. If decrease in time to recurrence is associated with long term patient benefits the cost-effectiveness evaluation will provide further evidence for the NHS to decide on full adoption of the technology.

For peer review only

For peer review only - http://bmjopen.bmj.com/site/about/guidelines.xhtml

4. List of abbreviations

CIS: Carcinoma in-situ

cm: centimetres

CT: Computerized tomography

DCIS: Ductal carcinoma in-site

EORTC: European Organization for Research and Treatment of Cancer

FFPE: Formalin Fixed Paraffin Embedded

HRQoL: Health related quality of life

HTA : health technology assessment

LCIS: Lobular carcinoma in situ

MIBC: Muscle invasive bladder cancer

MMC: Mitomycin-C

NHS: National Health Service

NIHR: National Institute for Health Research

NMIBC: Non-muscle invasive bladder cancer

PDD: Photo dynamic diagnosis

QALY: Quality adjusted life years

TURBT: Transurethral resection of bladder tumour

USS: Ultrasonography

WL: White light

For peer review only

Funding acknowledgement

This project was funded by the NIHR HTA (project number 11/142/02)

Department of Health disclaimer

The views expressed are those of the author(s) and not necessarily those of the NHS, the NIHR or the Department of Health.

The PHOTO trial is sponsored by Newcastle upon Tyne Hospitals NHS Foundation Trust.

Management of the study is divided between the Clinical Trials & Statistics Unit at the Institute of Cancer Research (ICR-CTSU) and Centre for Healthcare Randomised Trials (CHaRT) at the University of Aberdeen.

Independent Trial steering committee members – Mr Peter Whelan (Chair), Dr Andrew Vickers, Professor Per-Uno Malmstrom, Mr Allen Knight, Dr Dan Sjoberg.

Independent Data monitoring committee members – Dr Angela Casbard (Chair), Professor Diane Witham, Mr Robert Mills, Dr Ed Wilson, Mr Paul Silcocks.

For peer review only

Contributorship statement:

Zafer Tandogdu: conception and design of the work, drafting of the article, critical revision of the article and final approval of the version to be published

Rebecca Lewis: conception and design of the work, data collection, drafting of the article, critical revision of the article and final approval of the version to be published

Anne Duncan: conception and design of the work, data collection, drafting of the article, critical revision of the article and final approval of the version to be published

Alison McDonald: conception and design of the work, data collection, drafting of the article, critical revision of the article and final approval of the version to be published

Luke Vale: conception and design of the work, critical revision of the article and final approval of the version to be published

Steven Penegar: data collection, critical revision of the article and final approval of the version to be published

Jing Shen: design of the work, critical revision of the article and final approval of the version to be published

PHOTO TMG members: Trial management, data collection and final approval of the version to be published

Graeme Maclennan: design of the work, critical revision of the article and final approval of the version to be published

John Norrie: conception and design of the work, critical revision of the article and final approval of the version to be published

Emma Hall: conception and design of the work, critical revision of the article and final approval of the version to be published

Rakesh Heer: conception and design of the work, drafting of the article, critical revision of the article and final approval of the version to be published

For peer review only - http://bmjopen.bmj.com/site/about/guidelines.xhtml

For peer review only

For peer review only

For peer review only - http://bmjopen.bmj.com/site/about/guidelines.xhtml

5. References

1. UK, C.R. *Bladder cancer statistics*. Cancer statistics 2013 [cited 2016 05-09-2016]; Available from: http://www.cancerresearchuk.org/health-professional/cancer-statistics/statistics-by-cancer-type/bladder-cancer.
2. Epstein, J.I., et al., *The World Health Organization/International Society of Urological Pathology consensus classification of urothelial (transitional cell) neoplasms of the urinary bladder. Bladder Consensus Conference Committee.* Am J Surg Pathol, 1998. **22**(12): p. 1435-48.
3. Brausi, M., et al., *Variability in the recurrence rate at first follow-up cystoscopy after TUR in stage Ta T1 transitional cell carcinoma of the bladder: a combined analysis of seven EORTC studies.* Eur Urol, 2002. **41**(5): p. 523-31.
4. Bryan, R.T., et al., *Mechanisms of recurrence of Ta/T1 bladder cancer.* Ann R Coll Surg Engl, 2010. **92**(6): p. 519-24.
5. Babjuk, M., et al., *EAU Guidelines on Non-Muscle-invasive Urothelial Carcinoma of the Bladder: Update 2016.* Eur Urol, 2017. **71**(3): p. 447-461.
6. Mowatt, G., et al., *Photodynamic diagnosis of bladder cancer compared with white light cystoscopy: Systematic review and meta-analysis.* Int J Technol Assess Health Care, 2011. **27**(1): p. 3-10.
7. Sylvester, R.J., et al., *Predicting recurrence and progression in individual patients with stage Ta T1 bladder cancer using EORTC risk tables: a combined analysis of 2596 patients from seven EORTC trials.* Eur Urol, 2006. **49**(3): p. 466-5; discussion 475-7.
8. Rink, M., et al., *Hexyl Aminolevulinate-Guided Fluorescence Cystoscopy in the Diagnosis and Follow-up of Patients with Non-Muscle-invasive Bladder Cancer: A Critical Review of the Current Literature.* European urology, 2013. **64**(4): p. 624-38.
9. Sangar, V.K., et al., *The economic consequences of prostate and bladder cancer in the UK.* BJU Int, 2005. **95**(1): p. 59-63.
10. Leal, J., et al., *Economic Burden of Bladder Cancer Across the European Union.* Eur Urol, 2016. **69**(3): p. 438-47.
11. Yoshimura, K., et al., *Impact of superficial bladder cancer and transurethral resection on general health-related quality of life: an SF-36 survey.* Urology, 2005. **65**(2): p. 290-4.
12. Porter, M.P. and D.F. Penson, *Health related quality of life after radical cystectomy and urinary diversion for bladder cancer: a systematic review and critical analysis of the literature.* The Journal of urology, 2005. **173**(4): p. 1318-22.
13. *Comprehensive molecular characterization of urothelial bladder carcinoma.* Nature, 2014. **507**(7492): p. 315-22.
14. Miremami, J. and N. Kyprianou, *The promise of novel molecular markers in bladder cancer.* Int J Mol Sci, 2014. **15**(12): p. 23897-908.
15. Botteman, M.F., et al., *Quality of life aspects of bladder cancer: a review of the literature.* Qual Life Res, 2003. **12**(6): p. 675-88.
16. Miners, A., *Estimating 'costs' for cost-effectiveness analysis.* Pharmacoeconomics, 2008. **26**(9): p. 745-51.
17. Kaltenthaler, E., et al., *NICE Decision Support Unit Technical Support Documents*, in *NICE DSU Technical Support Document 13: Identifying and Reviewing Evidence to Inform the Conceptualisation and Population of Cost-Effectiveness Models*. 2011, National Institute for Health and Care Excellence (NICE)

unless otherwise stated. All rights reserved.: London.
18. Johnson, D.C., P.S. Greene, and M.E. Nielsen, *Surgical advances in bladder cancer: at what cost?* Urol Clin North Am, 2015. **42**(2): p. 235-52, ix.

For peer review only - http://bmjopen.bmj.com/site/about/guidelines.xhtml

19. van der Aa, M.N., et al., *Patients' perceived burden of cystoscopic and urinary surveillance of bladder cancer: a randomized comparison.* BJU Int, 2008. **101**(9): p. 1106-10.

6. Figure Legends:

Figure 1: White light (a) and blue light (photodynamic) (b) cystoscopy image of the bladder from a patient diagnosed with CIS. On PDD the area with CIS appears red whilst with WL the area is unclear.

Figure 2: PHOTO trial study design summary

For peer review only

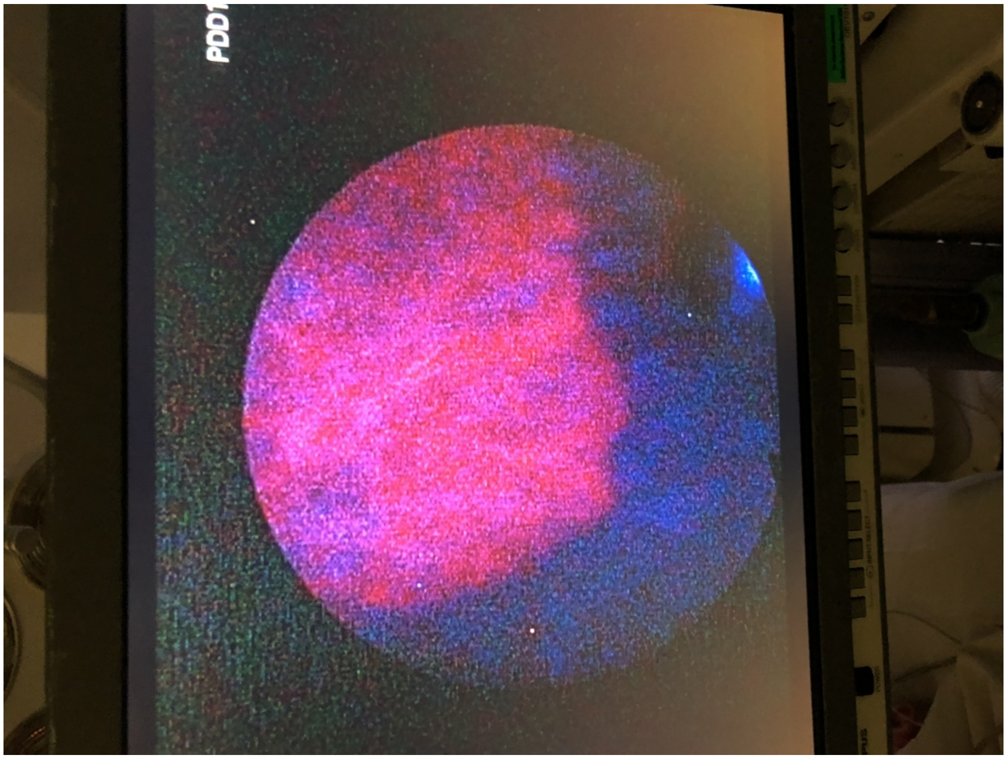

White light (a) and blue light (photodynamic) (b) cystoscopy image of the bladder from a patient diagnosed with CIS. On PDD the area with CIS appears red whilst with WL the area is unclear.

341x256mm (300 x 300 DPI)

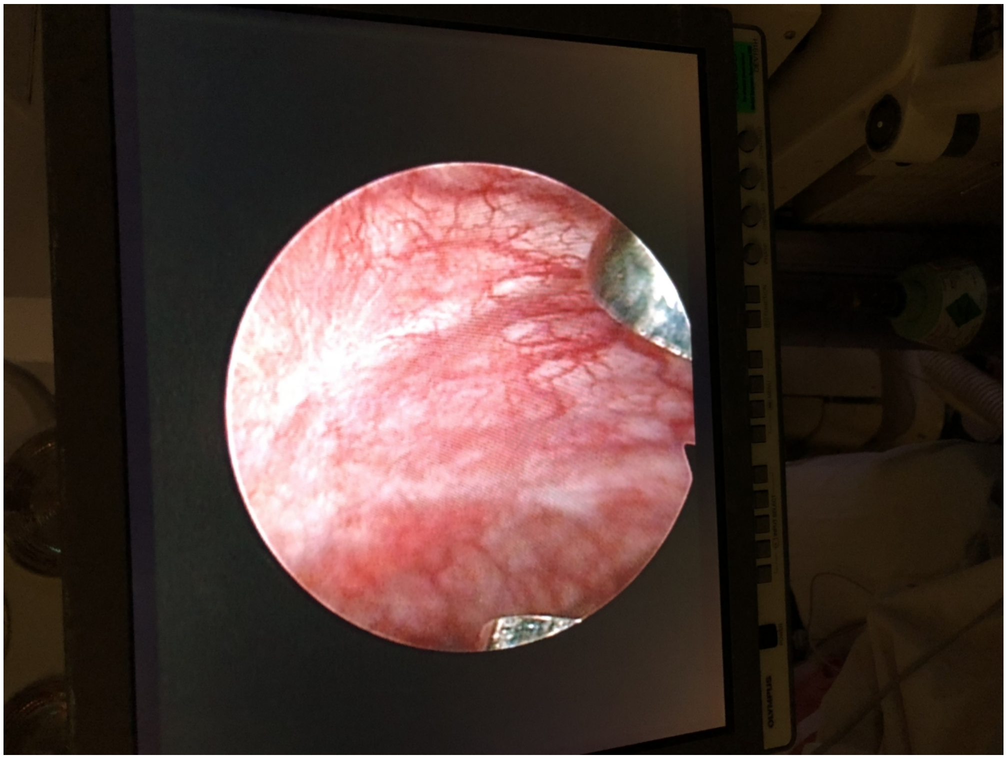

White light (a) and blue light (photodynamic) (b) cystoscopy image of the bladder from a patient diagnosed with CIS. On PDD the area with CIS appears red whilst with WL the area is unclear.

341x256mm (300 x 300 DPI)

APPENDIX-I: NICR PHOTO-T study

Sample Collection (per participant):

(a) 2 x blood samples totalling 12.5ml (10ml Streck blood collection tube for circulating DNA analysis and 2.5ml PAXgene blood collection tube for circulating RNA analysis);

(b) 2 x urine samples totalling 200ml (1 x 100ml for immediate translational processing and 1 x 100ml for biorepository storage), (c) 1 x FFPE core at baseline (plus core from recurrence, if occurs).

In total, 20 serially collected samples will be collected per PHOTO-T consented participant over the trial period (36 months), comprising 10 urine, 10 blood (5 DNA and 5 RNA) and 1 FFPE tumour tissue block. Collected at baseline (pre-treatment/TURBT) and treatment follow-up at 3, 12, 24 and 36 months or at recurrence (whichever comes first, predicted at 70% for the highest risk group over a trial period of 3 years).

Serial blood samples are requested for collection in clinic by research staff at participating investigator sites. Urine samples are provided at home by participants using specialist 'home collection' kits (home collection is preferable as sample quality in terms of genomic output is superior to clinically collected specimens) and FFPE blocks requested from Histopathology Departments of participating investigator sites retrospectively at the end of the trial.

For peer review only

BMJ Open

PHOTOdynamic versus white light-guided treatment of non-muscle invasive bladder cancer: A study protocol for a randomised trial of clinical and cost effectiveness

Journal:	*BMJ Open*
Manuscript ID	bmjopen-2018-022268.R2
Article Type:	Protocol
Date Submitted by the Author:	22-May-2019
Complete List of Authors:	tandogdu, zafer; Northern Institute for Cancer Research<br>Lewis, Rebecca; Institute of Cancer Research<br>Duncan, Anne; University of Aberdeen, Centre for Healthcare Randomised Trials (CHaRT)<br>McDonald, Alison; University of Aberdeen, Centre for Healthcare Randomised Trials (CHaRT)<br>Vale, Luke; Newcastle University, Health Economics Group, Institute of Health and Society<br>Penegar, Steven; Institute of Cancer Research<br>Shen, Jing; Institute of Health & Society<br>Kelly, John; University College London Hospitals NHS Foundation Trust, Urology<br>Pickard, Robert; Newcastle University Institute of Cellular Medicine<br>NDow, James; University of Aberdeen, Surgery<br>Ramsay, Craig; Health Service research unit, University of Aberdeen<br>Mostafid, Hugh; Hampshire Hospitals NHS Foundation Trust<br>Mariappan, Paramananthan; Western General Hospital, urology<br>Nabi, Ghulam; University of Dundee, Medicine<br>creswell, Joanne; South Tees Hospitals NHS Foundation Trust<br>Lazarowicz, Henry; Royal Liverpool and Broadgreen University Hospitals NHS Trust<br>McGrath, John; Royal Devon and Exeter NHS Foundation Trust, Urology<br>Taylor, Ernest; PHOTO Trial TMG<br>Clark, Emma; Northern Institute for Cancer Research<br>Maclennan, Graeme; University of Aberdeen, Centre for Healthcare Randomised Trials (CHaRT)<br>Norrie, John; Edinburgh Clinical Trials Unit, University of Edinburgh No. 9, Bioquarter<br>Hall, Emma; The Institute of Cancer Research<br>Heer, Rakesh; Northern Institute for Cancer Research
**Primary Subject Heading**:	Urology
Secondary Subject Heading:	Health economics, Oncology
Keywords:	bladder cancer, photodynamic diagnosis, quality of life, HEALTH ECONOMICS, Cost effectiveness, Sample biorepository

SCHOLARONE™
Manuscripts

Title page

PHOTOdynamic versus white light-guided treatment of non-muscle invasive bladder cancer: A study protocol for a randomised trial of clinical and cost effectiveness

Authors: Zafer Tandogdu[1](*), Rebecca Lewis[2](*), Anne Duncan[3](*), Alison McDonald[3], Luke Vale[4], Steven Penegar[2], Jing Shen[4], John D. Kelly[5](₸), Robert Pickard[6], James NDow[7](₸), Craig Ramsay[8](₸), Hugh Mostafid[9](₸), Paramananthan Mariappan [10](₸), Ghulam Nabi[11](₸), Joanne Creswell[12](₸), Henry Lazarowicz[13](₸), John McGrath[14](₸), Ernest Taylor[15](₸), Emma Clark[1](₸), Graeme Maclennan[3], John Norrie[16](**), Emma Hall[2](**), Rakesh Heer[1](**)

*Authors have provided equal contribution and are joint first authors.

**Joint senior authors.

₸ PHOTO Trial Management Group – to be listed as grouped authors.

1. Northern Institute for Cancer Research, Newcastle University, Newcastle upon tyne, UK
2. The Institute of Cancer Research, London, UK
3. Centre for Healthcare Randomised Trials (CHaRT), University of Aberdeen, Aberdeen, UK
4. Institute for Health and Society, Newcastle University, Newcastle upon Tyne, UK

For peer review only

For peer review only - http://bmjopen.bmj.com/site/about/guidelines.xhtml

5. University College London Hospitals NHS Foundation Trust, Urology Department, London, UK
6. Institute of Cellular Medicine, Newcastle University, UK
7. Academic Urology Unit, University of Aberdeen, Aberdeen, UK
8. Health Service Research Unit, University of Aberdeen, Aberdeen, UK
9. Basingstoke and North Hampshire NHS Foundation Trust, UK
10. Western General Hospital, Urology, Edinburgh, UK
11. University of Dundee, Medicine, Dundee, UK
12. South Tees Hospitals NHS Trust ,Middlesbrough, UK
13. Royal Liverpool and Broadgreen university Hospitals NHS Trust, UK
14. Royal Devon and Exeter Hospital NHS Trust, UK
15. PHOTO Trial patient representative, UK
16. Edinburgh Clinical Trials Unit, Edinburgh University, Edinburgh, UK

Corresponding authors:

1. Graeme Maclennan:
   - e-mail: G.maclennan@abdn.ac.uk
   - Phone:01224 438147

Key words:

- Bladder cancer
- Photodynamic diagnosis
- Quality of life
- Health economics
- Cost effectiveness

For peer review only - http://bmjopen.bmj.com/site/about/guidelines.xhtml

49 • Sample biorepository

50

51

52 The authors declare that they have no competing interests.

53

54

For peer review only

For peer review only - http://bmjopen.bmj.com/site/about/guidelines.xhtml

Abstract

Introduction: Bladder cancer is the most frequently occurring tumour of the urinary system. Ta, T1 tumours and carcinoma in situ (CIS) are grouped as non-muscle invasive bladder cancer (NMIBC), which can be effectively treated by transurethral resection of bladder tumour (TURBT). There are limitations to the visualisation of tumours with conventional TURBT using white light illumination within the bladder. Incomplete resections occur from the failure to identify satellite lesions or the full extent of the tumour leading to recurrence and potential risk of disease progression. To improve complete resection, photodynamic diagnosis (PDD) has been proposed as a method that can enhance tumour detection and guide resection. The objective of the current research is to determine whether PDD-guided TURBT is better than conventional white light surgery and whether it is cost-effective.

Methods and Analysis: PHOTO is a pragmatic multi-centre randomised controlled trial (open parallel group, non-masked, superiority trial) comparing the intervention of PDD-guided TURBT with standard white light resection in newly diagnosed intermediate and high risk NMIBC within the UK NHS setting. Clinical effectiveness is measured with time to recurrence. Cost-effectiveness is assessed within trial via the calculation of incremental cost per recurrence avoided and incremental cost per quality adjusted life per year (QALY) gained over three years, and over long term through a modelling exercise over patients' life time.

Ethics and dissemination: Formal ethics review was undertaken with a favourable opinion, in line with UK regulatory procedures (REC reference number: 14/NE/1062; Trial registration: ISRCTN84013636). If reductions in time to recurrence is associated with long term patient benefits the cost-effectiveness evaluation will provide further evidence to inform adoption of the technology. Findings will be

For peer review only - http://bmjopen.bmj.com/site/about/guidelines.xhtml

shared in lay media such as patient and charity forums and will be presented at key meetings and published in academic literature.

Article Summary:

Strengths and limitations of the PHOTO study include;

- the effectiveness of the photodynamic diagnosis (PDD) for the initial resection of intermediate and high risk non-muscle invasive bladder cancer as part of routine care will be demonstrated with a pragmatic clinical trial design,
- full-health economic evaluation will provide high quality evidence of the burden of NMIBC for the NHS
- a well-characterised trial associated biorepository of longitudinal serially collected tissue samples

For peer review only

For peer review only - http://bmjopen.bmj.com/site/about/guidelines.xhtml

1. Background

Bladder cancer is the most frequently occurring tumour of the urinary system [1]. Staging of bladder cancer is described using the Tumour, Node, Metastasis (TNM) system [2]. Tumours confined to the epithelial lining (urothelium) are classified as stage Ta and those invading the lamina propria are classified as stage T1.

Ta and T1 tumours can be removed by transurethral resection (TURBT) that involves passing a cystoscope through the urethra into the bladder and resecting the tumour under direct visualisation. For therapeutic purposes Ta and T1 tumours are grouped together as non-muscle invasive bladder cancer (NMIBC). Grade (microscopic characteristics of the tumour cells) can be used to describe aggressiveness of cancers and are characterised as either low grade (relatively benign) or high grade (aggressive). NMIBC also include flat, high-grade tumours that are confined to the epithelium classified as carcinoma in situ (CIS).

Complete resection of the tumour with TURBT is essential to obtain good prognosis. It is thought that failure to identify satellite tumours or to appreciate the full extent of the tumours visualised during resection using conventional white light cystoscopy may be a factor in 20-40% of recurrent bladder tumours [3, 4]. Incomplete resection with TURBT is also associated with staging errors. In order to correct the staging errors associated with initial TURBT a second resection within 2-6 weeks is suggested for select group patients [5]. It has been postulated that development in cystoscopy imaging can improve resections and decrease the need for a second resection [6].

Recurrence and stage progression to muscle invasive (T2-T4) or metastatic cancer is more likely to occur in those with high-grade tumours with concomitant

For peer review only - http://bmjopen.bmj.com/site/about/guidelines.xhtml

CIS. CIS in particular can be easily missed using conventional white light guided resection [6].

Surveillance of NMIBC is carried out with cystoscopy to detect recurrence early and allow treatment before progression. Clinical guidelines tailor follow up protocols according to the risk groups (low, intermediate and high) developed using clinical and histological parameters [7]. Advised follow-up of low risk is at three months and if negative the next cystoscopy is scheduled for nine months later and then yearly for five years. Patients with high-risk tumours have cystoscopy and urine cytology at three months. If negative, it is repeated every three months for two years, then every six months until five years, and annually thereafter [5]. The intensity of cystoscopic follow up for patients with intermediate risk is not clearly defined, for which a follow-up scheme in-between those described for low and high risk and is adapted according to personal and subjective factors [5].

1.1. Photodynamic diagnosis of NMIBC:

As an attempt to improve resection rates, photodynamic diagnosis (PDD) has been developed to enhance tumour detection and guide resection. A cystoscopy image of WL vs PDD is presented in figure 1. Meta-analyses and systematic reviews of PDD guided treatment of NMIBC have shown efficacy in tumour detection and reduction in residual tumour compared with white light cystoscopy alone. These findings translate into reduced recurrence rates [6, 8]. However, these trials were efficacy studies and the systematic review called for a pragmatic study to allow better interpretation of possible benefit into daily clinical practice.

1.2. Health economics of NMIBC:

For peer review only - http://bmjopen.bmj.com/site/about/guidelines.xhtml

NMIBC is one of the most costly cancers to manage on a per patient basis because of its high prevalence, high recurrence rate, need for adjuvant treatments and the requirement for long-term cystoscopic surveillance. The total cost of treatment and 5-year follow-up of patients with NMIBC diagnosed in the United Kingdom has increased from £73 million to £213 million from 2001 to 2012 (inflation corrected) [9, 10]. From a patient perspective, there often are considerable anxieties about recurrences, transurethral resection and progression, requiring additional therapies with potential mortality and long-term morbidity (e.g. radical surgery). Transurethral resection itself is associated with reduced quality of life, including both mental and physical health domains; although these effects are usually transient [11]. Substantial effects on health related quality of life (HRQoL) are most likely to come from adjuvant intravesical treatments and radical or palliative treatments for progression [12]. The cost-effectiveness of NMIBC treatment strategies has not been widely studied.

1.3. NMIBC biomarkers and clinical impact:

To date, existing non-invasive commercial biomarkers (primarily urinary) are not embedded in routine clinical practice due to poor sensitivity, specificity and lack of evidence. Several research bodies have recognized the lack of clinically useful biomarkers for bladder cancer. "Fit for purpose" sample resources accessible to high-throughput 'omic' technologies will afford the greatest opportunity to generate translational hypotheses and ensure clinical validity and utility of putative candidate markers/signatures [13, 14]. Robust, 'future-proof', longitudinal serial sample archives providing critical insights of the natural history of bladder cancer correlated with clinical detail for retrospective translational biomarker discovery, are lacking.

For peer review only - http://bmjopen.bmj.com/site/about/guidelines.xhtml

1.4. Current research objectives:

More efficient management strategies to reduce NMIBC recurrence and hence decrease both the burden to patients and costs are urgently needed. PDD-guided initial TURBT has been identified as a technique that can help achieve these aims. The objective of the current research (PHOTO trial) is to determine whether photodynamic surgery guided by a fluorescent tumour marker is better than conventional white light surgery in the cystoscopic treatment of people with intermediate and high risk cancers confined to the bladder lining and whether its implementation is cost-effective. The trial includes a full assessment of the costs of patient management through the care pathway. Individual patient data from this trial will be used for subsequent mathematical modelling studies to investigate safe monitoring frequency. The Photodynamic versus white light-guided treatment of non-muscle invasive bladder cancer trial has the following research objectives:

i. Primary objectives:

Clinical effectiveness: To compare time to recurrence for each of the two treatment strategies, with a principal point of interest at 3 years.

Cost-effectiveness: To evaluate cost-effectiveness as measured by the incremental cost per recurrence avoided and the cost-utility as measured by the incremental cost per quality-adjusted life year (QALY) gained at three years and over patients' life time.

ii. Secondary objectives:

a. To measure relative rates of disease progression at three years.
b. To measure relative harms and safety.
c. Patient lifetime HRQoL and cancer-specific survival.

For peer review only - http://bmjopen.bmj.com/site/about/guidelines.xhtml

iii. Additional objectives:

a. To model the safest and most cost-effective cystoscopic follow-up surveillance schedule;

b. To evaluate the learning curve for the procedure and account for its effects on outcomes of both PDD-guided and standard white light resections;

c. To establish a well-characterised cohort of serial samples from patients with intermediate and high-risk NMIBC including clinical data, urine, blood and tumour specimens for separately funded translational research.

2. Methods & Design

2.1. Study design

PHOTO is a multi-centre randomised open parallel group non-masked superiority trial comparing the intervention of PDD guided bladder tumour resection with standard white light resection in patients with newly diagnosed intermediate and high risk NMIBC. Apart from initial treatment, both groups will receive standard care, including single dose intravesical mitomycin C within 24 hours of initial resection, surveillance according to risk-adjusted schedules and adjuvant therapy as indicated by current practice guidelines. The target number of patients to be recruited is 533 with a trial specific follow-up of at least 36 months for each individual. The outline of the study protocol (Version 4, 02/02/2018) is shown in Figure 2.

For peer review only - http://bmjopen.bmj.com/site/about/guidelines.xhtml

2.2. Intervention

The interventions being compared within PHOTO trial are:

i. Photo dynamic diagnosis (PDD) guided trans-urethral resection (TURBT) (experimental group) vs;
ii. Standard white light TURBT (control group)

All participants, unless there are clinical contra-indications, receive intravesical mitomycin C (40 mg in 40 ml saline), ideally within 6 hours following TURBT but otherwise within the inpatient setting before discharge.

2.2.1. Technique of photodynamic diagnosis

PDD requires preliminary instillation of the photosensitiser hexaminolevulinate (85 mg in 50 ml of phosphate buffered saline) into the participant's bladder through a urethral catheter. Participants are asked not to pass urine for at least one hour after insertion. Following operating theatre preparation according to local standard procedures and under appropriate anaesthesia, participants undergo TURBT of their bladder tumour under blue light (wavelength 380-450 nm) illumination of the bladder. The equipment required includes a specialised light source, cystoscope, light cables and cameras. When using PDD, normal bladder epithelium appears blue whilst red areas should be considered suspicious and should be resected.

2.2.2. Technique of standard white light cystoscopy

The control group does not have any preliminary photosensitiser instillation and undergo standard tumour localisation and resection under white light (wavelength 400-800 nm) illumination of the bladder.

For peer review only - http://bmjopen.bmj.com/site/about/guidelines.xhtml

2.3. Participants

In the PHOTO trial adult patients with a suspected new diagnosis of intermediate or high risk NMIBC are studied. Participants are identified prior to initial resection based on results of preliminary visual assessment via cystoscopy or imaging performed as part of standard evaluation for suspected urinary tract malignancy.

Patients with the following criteria are included in the PHOTO trial:

- Adult men and women aged ≥ 16 years.
- First suspected diagnosis of bladder cancer.
- Visual/ultrasound/Computerized tomography (CT) diagnosis of intermediate/high risk NMIBC.
- White light visual appearances of intermediate or high risk disease (≥3cm OR two or more tumours OR flat velvety erythematous changes alerting a clinical suspicion of CIS).

  OR

  Suspicion of papillary bladder tumour ≥3cm based on ultrasound or CT scanning (without hydronephrosis).
- Written informed consent for participation prior to any study specific procedures.
- Willing to comply with the following life style guidelines:
  - Female participants must be surgically sterile or be post-menopausal, or must agree to use effective contraception after joining the study and for 7 days after treatment. Female participants must not breast feed for 7 days after treatment.

For peer review only

- Male participants must be surgically sterile or must agree to use effective contraception after joining the study and for 7 days after treatment.
- Effective contraception is defined as two forms of contraception, including one barrier method.

Exclusion criteria applied in the PHOTO trial are:

- Visual evidence of low risk NMIBC (solitary tumour < 3cm).
- Visual evidence of MIBC on preliminary cystoscopy, i.e. non-papillary or sessile mass (attached directly by its base without a stalk).
- Imaging evidence of MIBC – CT/USS (this includes the presence of hydronephrosis, which may be present despite clear imaging of MIBC in the bladder).
- Upper tract (kidney or ureteric) tumours on imaging.
- Any other malignancy in the past 2 years (except: non-melanomatous skin cancer cured by excision, adequately treated carcinoma in situ of the cervix, DCIS/LCIS of the breast or prostate cancer in patients who have a life expectancy of >5 years upon trial entry).
- Evidence of metastases.
- Porphyria or known hypersensitivity to porphyrins.
- Known pregnancy (based on history and without formal testing, in keeping with day-to-day NHS practice of PDD use).
- Any other conditions that in the Principal Investigator's opinion would contraindicate protocol treatment.
- Unable to provide informed consent.

For peer review only - http://bmjopen.bmj.com/site/about/guidelines.xhtml

- Unable or unwilling to complete follow-up schedule (including HRQoL questionnaires).

2.4. Informed consent-ethics approval

Favourable ethical opinion for this research was provided by the Newcastle & North Tyneside 2 ethics committee (REC reference number: 14/NE/1062) in July 2014. The study complies with the Helsinki Declaration and the principles of Good Clinical Practice (GCP).

Potential participants are identified mainly through rapid access haematuria clinics at participating sites from the UK. An eligibility checklist is completed by the local Principal Investigator (or delegate) to assess fulfilment of the entry criteria for all patients considered for the study. Information from the diagnostic cystoscopy is used to assess eligibility.

All potentially eligible patients are provided with an information sheet to explain why they have been approached and the nature of the study. Eligible patients are asked to provide written informed consent for the study only after they have had sufficient time to consider the trial and had the opportunity to have any further questions addressed.

2.5. Recruitment and randomisation

Eligible patients are centrally randomised using either the secure web-based or the 24-hour Interactive Voice Response randomisation system at the Centre for Healthcare Randomised Trials (CHaRT) in Aberdeen, using minimisation by centre and gender, to allocate participants 1:1 to the control and experimental groups. The minimisation algorithm incorporates a random element in order to prevent

For peer review only - http://bmjopen.bmj.com/site/about/guidelines.xhtml

313 deterministic treatment allocation. The trial was opened on the 23rd of October 2014 and recruitment was completed on 14th of February 2018. Over this period 22 centres joined the study and contributed to recruitment. List of sites participating are available on http://www.isrctn.com/ISRCTN84013636.

2.6. Outcome measures

2.6.1. Primary outcome measures

Clinical effectiveness: Time to recurrence is measured as time from randomisation to first recurrence.

Cost effectiveness: A health economic model will be developed to calculate incremental cost per recurrence avoided and incremental cost per QALY gained over three years.

2.6.2. Secondary outcome measures

Clinical effectiveness:

- Adverse events and complications up to 3 months from initial or second TURBT are captured and will be included in analysis.
- HRQOL is captured for each participant at baseline (prior to knowledge of treatment allocation), following surgery and at 3, 6, 12, 18, 24 and 36 months after randomisation.
- Disease progression is captured within the trial time. Patient life time projections will be made by using the trial data at baseline and supplementing as necessary from other published data.

For peer review only - http://bmjopen.bmj.com/site/about/guidelines.xhtml

- Overall survival and bladder cancer specific survival will be compared between the two treatment arms. Minimum follow-up of the last included patient will be 3 years and maximum expected follow-up is 66 months.

Cost effectiveness:

- Estimation of the incremental cost per recurrence avoided using the economic model over the patients' lifetime.
- Estimation of the incremental cost per QALY gained using the economic model over the patients' lifetime.

2.6.3. Additional outcomes measures

Schedules for follow-up: Using data from within the trial and, if appropriate, from other relevant sources, the risk of recurrence at each interval surveillance cystoscopy will be described to then model the most safe and efficient surveillance follow-up schedule.

The effect of PDD guided resection experience (learning curve) on clinical effectiveness: A subgroup analysis comparing outcomes from PDD-experienced and PDD-naïve surgeons (determined at baseline) will be conducted. Also for PDD-naïve surgeons an assessment of learning curve will be undertaken by comparing increasing experience and recurrence, in both PDD and WL resections.

2.7. Tracking and monitoring adverse events

Direct surgically related post-operative events occurring within 30 days following the first TURBT or second TURBT if required will be assessed using The Clavien Dindo classification for surgical complications. Events occurring up to 3 months after TURBT (second TURBT if required) will be assessed and recorded using the National

For peer review only - http://bmjopen.bmj.com/site/about/guidelines.xhtml

Cancer Institute Common Terminology Criteria for Adverse Events (CTCAE) v4.0 framework (http://ctep.cancer.gov/) [15].

2.8. Trial assessment and measures

PHOTO trial schedule of assessment and investigations are summarised in Table 1. Routine attendances for diagnosis and staging of new bladder cancers are used to establish eligibility, which includes obtaining the medical history. Eligible patients who consent for the trial are administered HRQoL questionnaires prior to primary TURBT and prior to discharge.

Time to recurrence will be measured from the day of randomisation to the day of subsequent biopsy with pathologically proven recurrence. Data will be collected from the following routine visits; 3, 6, 9, 12, 18, 24 and 36 months post initial TURBT (or second TURBT if required). Associated costs and changes in HRQoL will be measured. These will be collected by postal questionnaires sent directly to participants at 3, 6, 12, 18, 24 & 36 months post randomisation.

Disease progression will be assessed using results of further resection or imaging during follow-up. Progression will be defined as increase of stage into MIBC or development of nodal or metastatic disease. In addition, patients showing failure to respond to intravesical treatment (e.g. BCG failure) will also be captured.

The relative changes in HRQoL resulting from the physical and psychological benefit together with any harms associated with each strategy and with subsequent necessary cancer treatment will be measured using the generic EQ-5D-3L questionnaire, cancer specific EORTC-QLQ-C30 and disease-specific EORTC QLQ-NMIBC24 questionnaire completed by the participant.

For peer review only

For peer review only - http://bmjopen.bmj.com/site/about/guidelines.xhtml

Effect of PDD guided resection experience on clinical effectiveness: All recruiting surgeons will complete a learning curve questionnaire to elicit their white light and PDD resection experience prior to any recruitment. The subsequent accruing experience of each surgeon will be captured on case report forms. Early recurrence (12 weeks) will be used as a proxy of incomplete resection.

Table 1, Schedule of investigations/assessments in PHOTO trial

Visit/Assessment	Pre- randomisation screening	Pre-treatment	TURBT	Prior to discharge	Surveillance									At first disease recurrence/progression
					Second TURBT (as clinically indicated)	3 months post treatment	6 months post treatment	9 months post treatment	12 months post treatment	18 months post treatment	24 months post treatment	36 months post treatment	Annually thereafter	
Visual diagnosis of IR/HR NMIBC	X												According to EAU guidelines	Treatment according to local practice
Medical history	X													
HRQoL questionnaire[1]		X		X	X	X[2]	X[2]	X[2]	X[2]	X[2]	X[2]	X[2]		
TURBT according to treatment allocation with post treatment MMC instillation			X											
Second TURBT, if required, according to treatment allocation					X									
Assessment of adverse events (CTCAE & Clavien Dindo)						X								
Cystoscopy						X	X	X	X	X	X	X		
Histological confirmation of recurrence/ progression														X
Collection of FFPE tissue[3]			X											X
Urine sample collection[3]		X				X			X		X	X		X
Blood sample collection[3]		X				X			X		X	X		X

Footnotes

For peer review only - http://bmjopen.bmj.com/site/about/guidelines.xhtml

393 1. EORTC QLQ-C30 & NMIBC24, EQ-5D-3L
394 2. Questionnaire sent by-post directly to participant
395 3. If patient consented to participation in PHOTO-T (as this is archived pathology the tissue may be requested
396 at an interval from the diagnostic resection/recurrence).

For peer review only

For peer review only - http://bmjopen.bmj.com/site/about/guidelines.xhtml

2.9. Sample size

We aim to detect an absolute reduction in recurrence at three years of 12%; from 40% (under the conservative assumption that all the patients recruited are intermediate risk patients with a probability of recurrence of 0.4 at 3 years) to 28% (similar effect sizes of photodynamic therapy are reported in both intermediate and high risk groups) this will be equivalent to a relative reduction of 30%.

Recruitment of 533 participants (214 recurrences) will detect a hazard ratio of 0.64 between experimental and control strategies and provide, using the log-rank test, 90% power at a 2-sided 5% significance level. This calculation assumes 2.5 years incremental recruitment, a minimum of three years follow-up and a 6.4% follow-up attrition at end of year three. To achieve this we plan to use 30 secondary care sites that would see new bladder cancers diagnoses, from which we will exclude patients with MIBC (20%) and, from the remaining NMIBCs, exclude low risk disease (50%). Furthermore, we predict only 30% of these patients will be recruited based on willingness to participate or missed opportunities for recruitment.

2.10 Health economics analysis

A within trial cost-effectiveness analysis will be conducted to calculate incremental cost per recurrence avoided and incremental cost per QALY gained over three years. Data on costs, recurrence and QALYs for each participant will be recorded in the trial and used to estimate mean cost, recurrence and QALYs for each intervention group. As the time horizon of the trial is three years these data will be discounted at 3.5% [16]. The cost, recurrence avoided and QALY data will then be used to estimate incremental costs, recurrence avoided and QALYs and incremental cost per recurrence avoided and incremental costs per QALY.

For peer review only - http://bmjopen.bmj.com/site/about/guidelines.xhtml

An economic model will be developed to estimate relative rates of cost-effectiveness and cost-utility, at three years (to mirror the within trial analysis) and over a patient lifetime time horizon. An NHS perspective will be taken for the cost calculations. The model takes the form of a Markov state transition model that describes the consequences of different diagnosis and treatment strategies in terms of clinical and cost outcomes [6]. The rates of recurrence and progression recorded with the 3-year follow-up of the trial will be used to model short-term recurrence and progression rates. Further data required for the model relates to the transition and other probabilities of events beyond the 3 year follow-up, including the risk of recurrence and progression, probabilities of receiving different types of intervention should progression or recurrences occur, and risks of mortality (both from bladder cancer and other causes), will be sought through a structured systematic review of long-term outcomes of treatments of bladder cancer. The model will be used to produce estimates of costs, QALYs, recurrence rates and survival. Both costs and outcomes will be discounted at 3.5% in the base case analyses. Cost-effectiveness will be reported as incremental cost per QALY gained and incremental cost per recurrence avoided (at both 3 years and over the patient's lifetime). These data will be presented as point estimates and bootstrapping techniques will be used to estimate the statistical imprecision surrounding them. The results of this stochastic analysis will be presented as cost and QALY plots and as cost-effectiveness acceptability curves. Further deterministic sensitivity analyses will be conducted to explore other forms of uncertainty e.g. surrounding the choice of discount rate or around the unit costs of equipment. The model will be probabilistic and distributions will be attached to all

For peer review only - http://bmjopen.bmj.com/site/about/guidelines.xhtml

parameters, the shape and type of distribution will depend upon the data available and recommendations for good practice in modelling [17].

2.10. Patient and Public Involvement

Patient involvement was ensured at the early stages of protocol development and contributed to user-lead development of outcomes of value to patients in the design of the trial. Additionally, the patient journey of patient representatives was investigated through the diagnosis and treatment of bladder cancer, which includes an anonymised account impact on his quality of life. This helped understand the burden of the intervention on patients. A patient representative was involved as a co-investigator and member of the trial steering committee helping manage and analyse the implications of the research.

2.11. PHOTO-Translational side study

PHOTO Translational (PHOTO-T) aims to establish a well-characterised trial associated biorepository of longitudinal serially collected tissue samples (blood, urine and FFPE). The collection of samples from PHOTO patients is optional with every PHOTO-T consented participant collecting a urine and blood sample at baseline (pre-treatment/TURBT) and 3, 12, 24 and 36 months treatment follow-up or at recurrence (whichever comes first, predicted at 70% for the highest risk group over a trial period of 3 years). A FFPE core at baseline (plus recurrence, if occurs) will also be collected (supplement).

3. Discussion

Bladder cancer is the most frequent urothelial cancer and the overall costs for treatment and follow-up remain higher than most other cancers [18]. Achieving complete resection of NMIBC with TURBT is associated with lower recurrence rates

For peer review only

For peer review only - http://bmjopen.bmj.com/site/about/guidelines.xhtml

in follow-up. However, it is unclear if this translates into lower progression rates in long-term follow-up. PDD guided initial TURBT is a technology that could improve resection and ultimately reduce recurrence and the need for further treatments. Studies on PDD have demonstrated the efficacy of the technology using strict study entry requirements, for which translation into daily clinical practice is limited. Therefore, in the PHOTO trial the effectiveness of the technology as part of routine care will be demonstrated with a pragmatic clinical trial design.

PHOTO trial includes measurement of HRQoL using EQ-5D at the time of initial treatment and surveillance. The measurement of HRQoL around the time of the cystoscopy and TURBT can be particularly dynamic due to an acute deterioration in health score associated with the invasive procedure followed by a typical rapid recovery [11, 19]. Therefore, a side study was developed, where patients are recruited from the PHOTO trial to evaluate the acute deterioration in quality of life by suspected diagnosis or TURBT around the time of resection. This side study will use a time trade off exercise and the outcomes will supplement the calculation of QALYs in the health economic model.

The high costs of bladder cancer to health care systems has usually been obtained from weak data and the true costs are unclear. The pragmatic design of the PHOTO trial alongside the robust data collection for a full-health economic evaluation will provide high quality evidence of the burden of NMIBC for the NHS. Moreover, it will also provide a cost effectiveness comparison of white light vs PDD-guided initial TURBT resections.

Evidence on the required cases for PDD naïve surgeons to gain competency the technology is weak. This could act as a potential confounder on the clinical outcomes measured and therefore will be accounted for during analysis. Moreover, an

For peer review only - http://bmjopen.bmj.com/site/about/guidelines.xhtml

evaluation of the learning curve of PDD will also be carried out using the forms filled in by surgeons.

The primary outcome of the study is time to recurrence measured from the day of randomisation to the day of subsequent biopsy with pathologically proven recurrence. If decrease in time to recurrence is associated with long term patient benefits the cost-effectiveness evaluation will provide further evidence for the NHS to decide on full adoption of the technology.

For peer review only

For peer review only - http://bmjopen.bmj.com/site/about/guidelines.xhtml

4. List of abbreviations

CIS: Carcinoma in-situ

cm: centimetres

CT: Computerized tomography

DCIS: Ductal carcinoma in-site

EORTC: European Organization for Research and Treatment of Cancer

FFPE: Formalin Fixed Paraffin Embedded

HRQoL: Health related quality of life

HTA : health technology assessment

LCIS: Lobular carcinoma in situ

MIBC: Muscle invasive bladder cancer

MMC: Mitomycin-C

NHS: National Health Service

NIHR: National Institute for Health Research

NMIBC: Non-muscle invasive bladder cancer

PDD: Photo dynamic diagnosis

QALY: Quality adjusted life years

TURBT: Transurethral resection of bladder tumour

USS: Ultrasonography

WL: White light

For peer review only

Funding acknowledgement

This project was funded by the NIHR HTA (project number 11/142/02)

Department of Health disclaimer

The views expressed are those of the author(s) and not necessarily those of the NHS, the NIHR or the Department of Health.

The PHOTO trial is sponsored by Newcastle upon Tyne Hospitals NHS Foundation Trust.

Management of the study is divided between the Clinical Trials & Statistics Unit at the Institute of Cancer Research (ICR-CTSU) and Centre for Healthcare Randomised Trials (CHaRT) at the University of Aberdeen.

Independent Trial steering committee members – Mr Peter Whelan (Chair), Dr Andrew Vickers, Professor Per-Uno Malmstrom, Mr Allen Knight, Dr Dan Sjoberg.

Independent Data monitoring committee members – Dr Angela Casbard (Chair), Professor Diane Witham, Mr Robert Mills, Dr Ed Wilson, Mr Paul Silcocks.

For peer review only

For peer review only - http://bmjopen.bmj.com/site/about/guidelines.xhtml

Contributorship statement:

Zafer Tandogdu: conception and design of the work, drafting of the article, critical revision of the article and final approval of the version to be published

Rebecca Lewis: conception and design of the work, data collection, drafting of the article, critical revision of the article and final approval of the version to be published

Anne Duncan: conception and design of the work, data collection, drafting of the article, critical revision of the article and final approval of the version to be published

Alison McDonald: conception and design of the work, data collection, drafting of the article, critical revision of the article and final approval of the version to be published

Luke Vale: conception and design of the work, critical revision of the article and final approval of the version to be published

Steven Penegar: data collection, critical revision of the article and final approval of the version to be published

Jing Shen: design of the work, critical revision of the article and final approval of the version to be published

PHOTO TMG members: Trial management, data collection and final approval of the version to be published

Graeme Maclennan: design of the work, critical revision of the article and final approval of the version to be published

John Norrie: conception and design of the work, critical revision of the article and final approval of the version to be published

Emma Hall: conception and design of the work, critical revision of the article and final approval of the version to be published

Rakesh Heer: conception and design of the work, drafting of the article, critical revision of the article and final approval of the version to be published

For peer review only

For peer review only - http://bmjopen.bmj.com/site/about/guidelines.xhtml

573

For peer review only

For peer review only - http://bmjopen.bmj.com/site/about/guidelines.xhtml

5. References

1. UK, C.R. *Bladder cancer statistics*. Cancer statistics 2013 [cited 2016 05-09-2016]; Available from: http://www.cancerresearchuk.org/health-professional/cancer-statistics/statistics-by-cancer-type/bladder-cancer.
2. Epstein, J.I., et al., *The World Health Organization/International Society of Urological Pathology consensus classification of urothelial (transitional cell) neoplasms of the urinary bladder. Bladder Consensus Conference Committee.* Am J Surg Pathol, 1998. **22**(12): p. 1435-48.
3. Brausi, M., et al., *Variability in the recurrence rate at first follow-up cystoscopy after TUR in stage Ta T1 transitional cell carcinoma of the bladder: a combined analysis of seven EORTC studies.* Eur Urol, 2002. **41**(5): p. 523-31.
4. Bryan, R.T., et al., *Mechanisms of recurrence of Ta/T1 bladder cancer.* Ann R Coll Surg Engl, 2010. **92**(6): p. 519-24.
5. Babjuk, M., et al., *EAU Guidelines on Non-Muscle-invasive Urothelial Carcinoma of the Bladder: Update 2016.* Eur Urol, 2017. **71**(3): p. 447-461.
6. Mowatt, G., et al., *Photodynamic diagnosis of bladder cancer compared with white light cystoscopy: Systematic review and meta-analysis.* Int J Technol Assess Health Care, 2011. **27**(1): p. 3-10.
7. Sylvester, R.J., et al., *Predicting recurrence and progression in individual patients with stage Ta T1 bladder cancer using EORTC risk tables: a combined analysis of 2596 patients from seven EORTC trials.* Eur Urol, 2006. **49**(3): p. 466-5; discussion 475-7.
8. Rink, M., et al., *Hexyl Aminolevulinate-Guided Fluorescence Cystoscopy in the Diagnosis and Follow-up of Patients with Non-Muscle-invasive Bladder Cancer: A Critical Review of the Current Literature.* European urology, 2013. **64**(4): p. 624-38.
9. Sangar, V.K., et al., *The economic consequences of prostate and bladder cancer in the UK.* BJU Int, 2005. **95**(1): p. 59-63.
10. Leal, J., et al., *Economic Burden of Bladder Cancer Across the European Union.* Eur Urol, 2016. **69**(3): p. 438-47.
11. Yoshimura, K., et al., *Impact of superficial bladder cancer and transurethral resection on general health-related quality of life: an SF-36 survey.* Urology, 2005. **65**(2): p. 290-4.
12. Porter, M.P. and D.F. Penson, *Health related quality of life after radical cystectomy and urinary diversion for bladder cancer: a systematic review and critical analysis of the literature.* The Journal of urology, 2005. **173**(4): p. 1318-22.
13. *Comprehensive molecular characterization of urothelial bladder carcinoma.* Nature, 2014. **507**(7492): p. 315-22.
14. Miremami, J. and N. Kyprianou, *The promise of novel molecular markers in bladder cancer.* Int J Mol Sci, 2014. **15**(12): p. 23897-908.
15. Botteman, M.F., et al., *Quality of life aspects of bladder cancer: a review of the literature.* Qual Life Res, 2003. **12**(6): p. 675-88.
16. Miners, A., *Estimating 'costs' for cost-effectiveness analysis.* Pharmacoeconomics, 2008. **26**(9): p. 745-51.
17. Kaltenthaler, E., et al., *NICE Decision Support Unit Technical Support Documents*, in *NICE DSU Technical Support Document 13: Identifying and Reviewing Evidence to Inform the Conceptualisation and Population of Cost-Effectiveness Models*. 2011, National Institute for Health and Care Excellence (NICE)

unless otherwise stated. All rights reserved.: London.
18. Johnson, D.C., P.S. Greene, and M.E. Nielsen, *Surgical advances in bladder cancer: at what cost?* Urol Clin North Am, 2015. **42**(2): p. 235-52, ix.

For peer review only - http://bmjopen.bmj.com/site/about/guidelines.xhtml

19. van der Aa, M.N., et al., *Patients' perceived burden of cystoscopic and urinary surveillance of bladder cancer: a randomized comparison.* BJU Int, 2008. **101**(9): p. 1106-10.

6. Figure Legends:

Figure 1: White light (a) and blue light (photodynamic) (b) cystoscopy image of the bladder from a patient diagnosed with CIS. On PDD the area with CIS appears red whilst with WL the area is unclear.

Figure 2: PHOTO trial study design summary

For peer review only

For peer review only - http://bmjopen.bmj.com/site/about/guidelines.xhtml

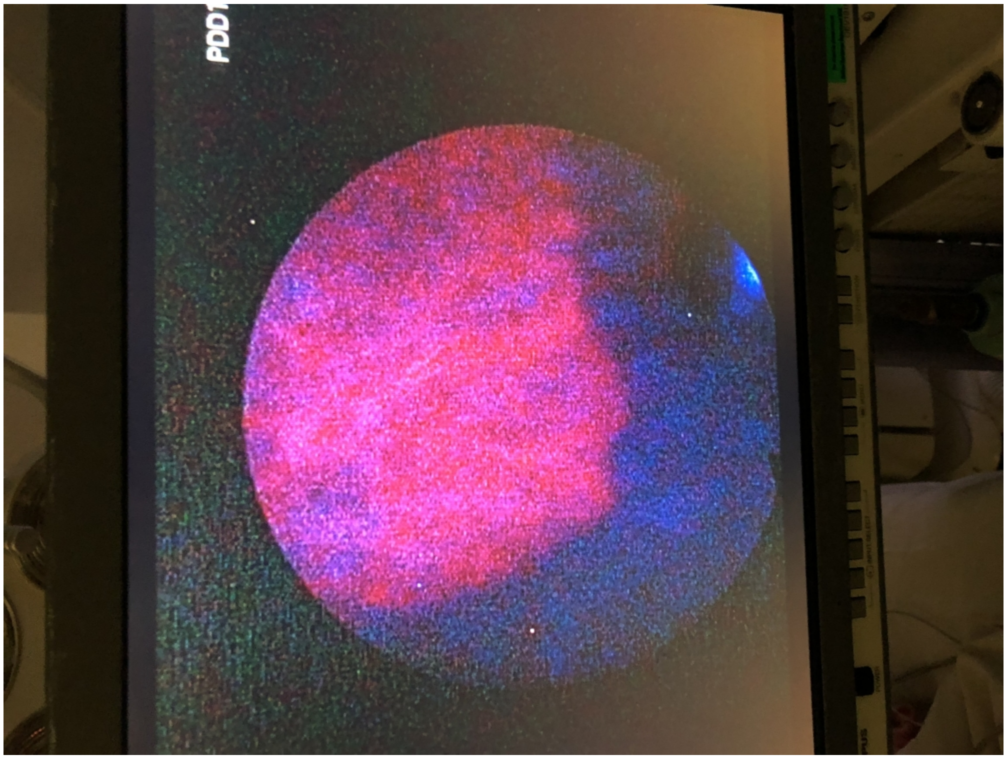

White light (a) and blue light (photodynamic) (b) cystoscopy image of the bladder from a patient diagnosed with CIS. On PDD the area with CIS appears red whilst with WL the area is unclear.

341x256mm (300 x 300 DPI)

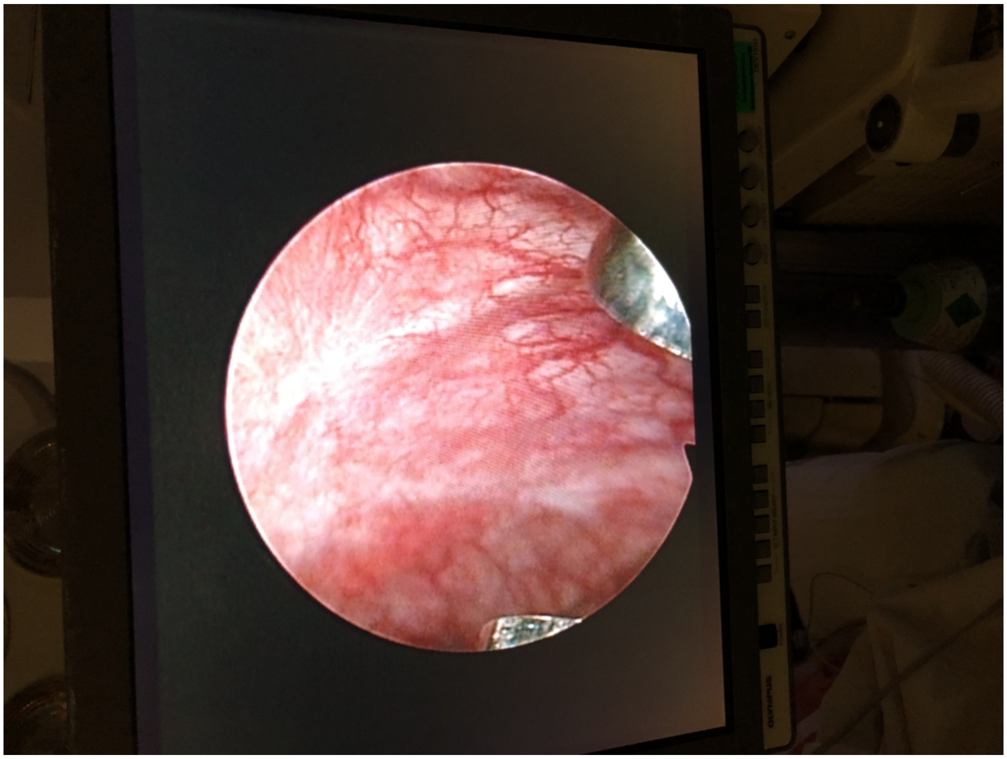

White light (a) and blue light (photodynamic) (b) cystoscopy image of the bladder from a patient diagnosed with CIS. On PDD the area with CIS appears red whilst with WL the area is unclear.

341x256mm (300 x 300 DPI)

APPENDIX-I: NICR PHOTO-T study

Sample Collection (per participant):

(a) 2 x blood samples totalling 12.5ml (10ml Streck blood collection tube for circulating DNA analysis and 2.5ml PAXgene blood collection tube for circulating RNA analysis);

(b) 2 x urine samples totalling 200ml (1 x 100ml for immediate translational processing and 1 x 100ml for biorepository storage), (c) 1 x FFPE core at baseline (plus core from recurrence, if occurs).

In total, 20 serially collected samples will be collected per PHOTO-T consented participant over the trial period (36 months), comprising 10 urine, 10 blood (5 DNA and 5 RNA) and 1 FFPE tumour tissue block. Collected at baseline (pre-treatment/TURBT) and treatment follow-up at 3, 12, 24 and 36 months or at recurrence (whichever comes first, predicted at 70% for the highest risk group over a trial period of 3 years).

Serial blood samples are requested for collection in clinic by research staff at participating investigator sites. Urine samples are provided at home by participants using specialist 'home collection' kits (home collection is preferable as sample quality in terms of genomic output is superior to clinically collected specimens) and FFPE blocks requested from Histopathology Departments of participating investigator sites retrospectively at the end of the trial.

For peer review only

SPIRIT
STANDARD PROTOCOL ITEMS: RECOMMENDATIONS FOR INTERVENTIONAL TRIALS

SPIRIT 2013 Checklist: Recommended items to address in a clinical trial protocol and related documents*

Section/item	Item No	Description
**Administrative information**		
Title	1	2-4
Trial registration	2a	78
	2b	supplement
Protocol version	3	215
Funding	4	532
Roles and responsibilities	5a	549-573
	5b	538
	5c	541-547
	5d	541-547
**Introduction**		
Background and rationale	6a	97-170
	6b	207-224
Objectives	7	185-203
Trial design	8	206-215, 309-316
**Methods: Participants, interventions, and outcomes**		
Study setting	9	297-301. http://www.isrctn.com/ISRCTN84013636 https://doi.org/10.1186/ISRCTN84013636
Eligibility criteria	10	243-290
Interventions	11a	227-235
	11b	Not applicable

For peer review only

	11c	Not applicable
	11d	Not applicable
Outcomes	12	321-356
Participant timeline	13	367-397
Sample size	14	398-412
Recruitment	15	450-457
**Methods: Assignment of interventions (for controlled trials)**		
Allocation:		
Sequence generation	16a	311-314
Allocation concealment mechanism	16b	311-314
Implementation	16c	309-311
Blinding (masking)	17a	Not applicable – surgical intervention
	17b	Not applicable – surgical intervention
**Methods: Data collection, management, and analysis**		
Data collection methods	18a	367-397
	18b	Protocol
Data management	19	Protocol
Statistical methods	20a	415-448 + Protocol
	20b	Protocol
	20c	Protocol
**Methods: Monitoring**		
Data monitoring	21a	546-547 & Protocol
	21b	Protocol
Harms	22	359-364
Auditing	23	Protocol

Ethics and dissemination

Research ethics approval	24	76-78 & 293-294
Protocol amendments	25	78-82
Consent or assent	26a	297-300
	26b	461-462
Confidentiality	27	protocol
Declaration of interests	28	No conflict of interests
Access to data	29	Protocol
Ancillary and post-trial care	30	Protocol
Dissemination policy	31a	Protocol
	31b	Protocol
	31c	Protocol

Appendices

Informed consent materials	32	Protocol
Biological specimens	33	459-465 & supplement

*It is strongly recommended that this checklist be read in conjunction with the SPIRIT 2013 Explanation & Elaboration for important clarification on the items. Amendments to the protocol should be tracked and dated. The SPIRIT checklist is copyrighted by the SPIRIT Group under the Creative Commons "Attribution-NonCommercial-NoDerivs 3.0 Unported" license.

For peer review only
